# Palladium catalyst immobilized on functionalized microporous organic polymers for C–C coupling reactions†

**DOI:** 10.1039/c9ra07303e

**Published:** 2019-10-28

**Authors:** Wei Xu, Cijie Liu, Dexuan Xiang, Qionglin Luo, You Shu, Hongwei Lin, Yangjian Hu, Zaixing Zhang, Yuejun Ouyang

**Affiliations:** Hunan Engineering Laboratory for Preparation Technology of Polyvinyl Alcohol (PVA) Fiber Material, Institute of Organic Synthesis, Huaihua University Huaihua 418000 China dexuanxiang@126.com oyyj0816@163.com

## Abstract

Two microporous organic polymer immobilized palladium (MOP-Pd) catalysts were prepared from benzene and 1,10-phenanthroline by Scholl coupling reaction and Friedel–Crafts reaction, respectively. The structure and composition of the catalyst were characterized by FT-IR, TGA, N_2_ sorption, SEM, TEM, ICP-AES and XPS. MOP-Pd catalysts were found to possess high specific surface areas, large pore volume and low skeletal bone density. Moreover, the immobilized catalyst also had advantages, such as readily available raw materials, chemical and thermal stability, and low synthetic cost. The Pd catalyst is an effective heterogeneous catalyst for carbon–carbon (C–C) coupling reactions, such as the Heck reaction and Suzuki–Miyaura reaction, affording good to high yields. In these reactions, the catalyst was easily recovered and reused five times without significant activity loss.

## Abstract 2

Two microporous organic polymers were prepared from 1,10-phenanthroline by Scholl coupling reaction and Friedel–Crafts reaction, and applied to Heck reaction and Suzuki–Miyaura reaction as heterogeneous catalysts.

## Graphical abstract

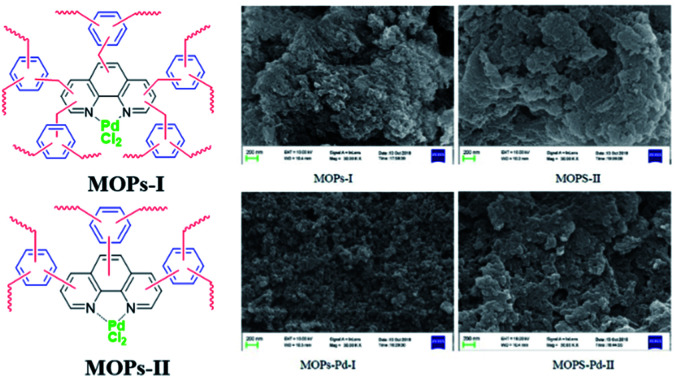

Carbon–carbon (C–C) coupling reactions have become one of the most versatile and utilized reactions for the selective construction of C–C bonds for the formation of functionalised aromatics,^1^ natural products,^2^ pharmaceuticals,^3^ polymers^4^ and advanced materials.^5^ Many transition metals have been used as catalysts in these reactions, aided by a great variety of ligands ranging from simple, commercial phosphines to complex custom-made molecules.^6^ Among these transition metals, palladium plays a significant role in various cross-coupling reactions, such as Suzuki,^7^ Heck,^8^ Sonogashira,^9^ Stille,^10^ and Ullmann coupling reactions,^11^ due to their strong electrical and chemical properties.^12^ Over the past decades, various homogeneous catalytic systems have been developed for organic transformations,^13^ which often progress smoothly under the inert atmosphere in organic solvents, for example, toluene or tetrahydrofuran in the presence of soluble palladium complexes as catalysts. However, most homogeneous palladium catalysts suffer from drawbacks such as high-cost of phosphine ligands, use of various additives, difficult separation, metal leaching, recovery, recyclability, and the toxicity of phosphine ligands.

Heterogeneous catalysis have attracted increasing attention as they have been proven to be useful for different organic reactions owning to their unique properties, such as high reactivity, stability, easy separation, purification and recyclability.^14^ Many active heterogeneous palladium catalysts have been developed and widely applied in the C–C coupling reactions.^15^ Palladium has been immobilized on various solid supporting materials, such as zeolite,^16^ silica,^17^ metal organic frameworks,^18^ and functionalized graphene oxide.^19^ However, a substantial decrease in activity and selectivity of the heterogeneous palladium catalysts is frequently observed because of their long diffusion pathway to catalytic sites and the difference of electron density on active sites. To address these problems, materials with larger interface and more active site are employed to support palladium as heterogeneous catalysts, such as palladium immobilized on hyper-crosslinked polymers were high activity in Suzuki–Miyaura coupling reaction.^20^

Microporous organic polymers (MOPs) consists of purely organic elements have recently emerged as versatile platforms for heterogeneous catalysts thanks to their unique properties, including superior chemical, thermal and hydrothermal stability, synthetic diversity, low skeletal density and high surface area.^20,21^ More importantly, the bottom–up approach of MOPs provides an opportunity for the design of polymer frameworks with a range of functionalities into the porous structure to use as catalysts or ligands.^22^ Recently, Kaskel reported the incorporation of a thermally fragile imidazolium moiety into MOPs resulted in a heterogeneous organocatalyst active in carbene-catalyzed Umpolung reaction.^23^ Wang designed photocatalysts with microporous *via* the copolymerization from pyrene and dibenzothiophene-S,S-dioxide building blocks and tested the effect of the photocatalytic hydrogen evolution.^24^ Xu described the synthesis of microporous with N-heterocyclic carbenes by an external cross-linking reaction and applied it in Suzuki reaction.^25^ Zhou demonstrated for the first time that the microporous structure has a positive effect on controlling selectivities in the hydrosilylation of alkynes.^26^ Recently, we also reported three pyridine-functionalized N-heterocyclic carbene–palladium complexes and its application in Suzuki–Miyaura coupling reactions.^27^

1,10-phenanthroline is an ideal candidate of ligands due to its structural features such as two N-atom placed in juxta position to provide binding sites for metal cations.^28^ To utilize the unique structure feature, we employed it in the construction of MOPs *via* Scholl and Friedel–Crafts reaction, respectively. Therefore, this paper presents our recent studies on the synthesis of two heterogeneous palladium catalysts supported on MOPs through a simple and low-cost procedure. These catalysts displayed remarkable catalytic activity in C–C coupling reactions, including Suzuki–Miyaura reaction and Heck coupling reaction. The properties of simple preparation, wide application of this catalyst and good performance in C–C coupling reactions and adaptability with various substrates make it perfect catalytic option for C–C coupling reactions.

The microporous network with 1,10-phenanthroline functional groups and incorporation of Pd metal were confirmed by Fourier transform infrared (FT-IR) spectroscopy. The FT-IR spectra of MOPs and MOPs-Pd (Fig. 1) displayed a series of bands around 2800–3100 cm^−1^, which were assigned to the C–H stretching band and in-of-plane bending vibrations of the aryl rings. The bands around 1550–1750 cm^−1^ were attributed to the –C=N- stretching band. The bands around 1400–1450 and 850–700 cm^−1^ were corresponded to the benzene and 1,10-phenanthroline skeletal stretching and the C–H out-of-plane bending vibrations of the aryl rings, respectively. The bond around 1495 cm^−1^ in MOPs-I and MOPs-Pd-I is assigned to in-of-plane bending vibrations of CH_2_, which indicated that 1,10-phenanthroline and benzene were linked by CH_2_.

**Fig. 1:**
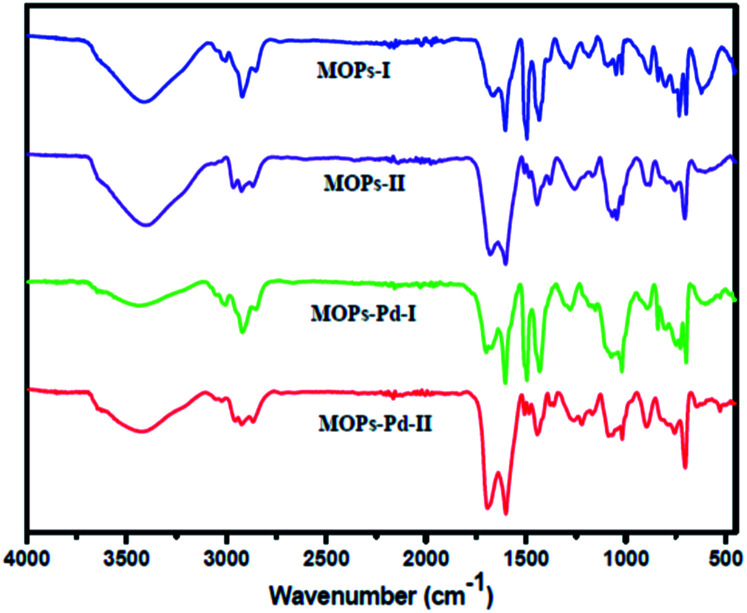
FT-IR spectra of MOPs and MOPs-Pd.

The X-ray photoelectron spectroscopy (XPS) analysis of the MOPs-Pd is performed to investigate the coordination states of palladium species (Fig. 2). In Fig. 2, the Pd 3d XPS spectra of the MOPs-Pd-I catalysts reveal that Pd is present in the +2 oxidation state rather than in the metallic state. This is corresponding to the binding energy (B.E.) of 337.4 eV and 342.4 eV, which are assigned to be Pd 3d_5/2_ and 3d_3/2_ of Pd (+2), respectively. Compared with the PdCl_2_ (337.9 eV and 343.1 eV), the Pd^2+^ binding energy in the MOPs-Pd-I catalyst shifts negatively by 0.5 eV and 0.7 eV. This can be attributed to the effect of the coordination with 1,10-phenanthroline in microporous networks. The results show that Pd^2+^ can be immobilized successfully on the MOPs by coordinating to 1,10-phenanthroline rather than by physical adsorption of Pd^2+^ on the surface. XPS graphs of MOPs-Pd-II also reveal that Pd^2+^ is immobilized on MOPs materials.

**Fig. 2:**
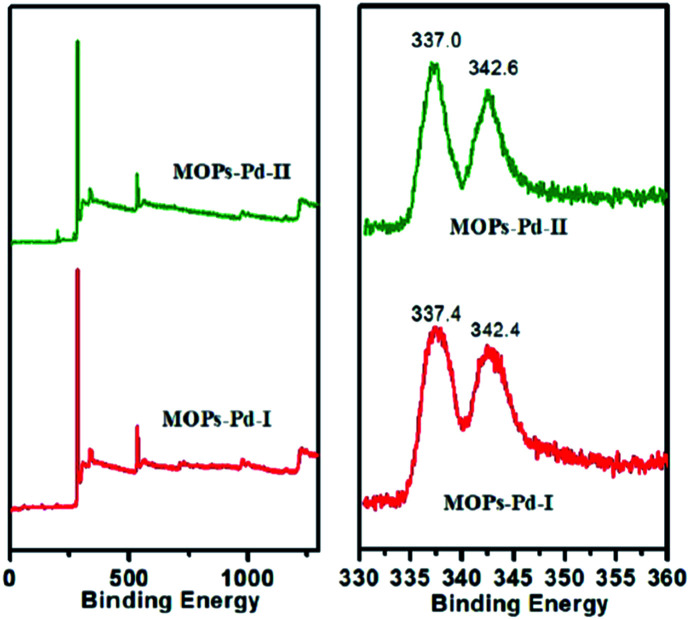
XPS spectra of the MOPs-Pd.

The surface area and pore structure of the MOPs and MOPs-Pd were investigated by nitrogen adsorption analyses at 77.3 K. In Fig. 3, the MOPs-Pd exhibits type I adsorption–desorption isotherms, which is similar to the isotherms exhibited by the parent MOPs polymers. The result implies that these microporous organic polymers and metalized polymers consist of both micropores and mesopores. The apparent Brunauer–Emmett–Teller surface areas (*S*_BET_) of MOPs-Pd are smaller than those of the non-metallized parent networks (Table 1), which can be attributed to both the partial pore filling with metal and simple increase in mass. However, the materials are still significantly microporous because of many micropore surface (*S*_Micro_) and micropore volume (*V*_Micro_) in these materials. The abundant micropores with a suitable size favored naturally the dispersion of the metal species. The presence of a spot of mesopore and macropore structure in the heterogeneous catalyst is also essential, because these structures enabled the frameworks to be highly soaked in a certain solvent. As a result, the accessibility of the catalytically active sites toward the substrates is maximized. The ICP-AES analysis indicates that the content of Pd in MOPs-Pd I and II are 2.5 and 2.4 wt% (Table 1), respectively.

**Fig. 3:**
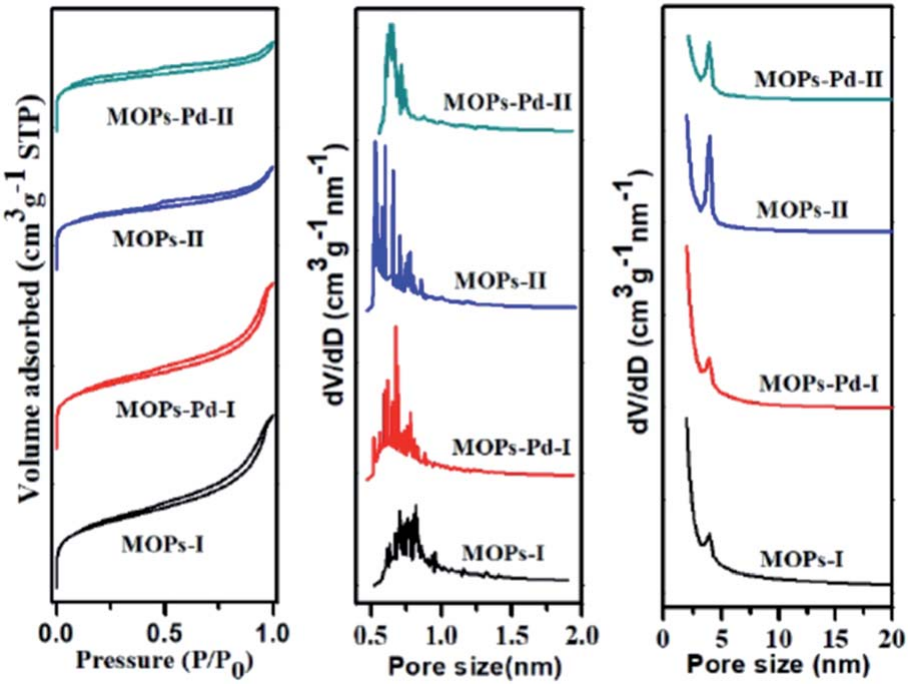
N_2_ adsorption–desorption isotherms and corresponding pore size distributions of MOPs and MOPs-Pd.

**Table 1:** Physical properties of MOPs and MOPs-Pd

Sample	*S*_BET_ a [m^2^ g^−1^]	*S*_Micro_ b [m^2^ g^−1^]	V_Micro_^c^ [m^3^ g^−1^]	[Pd]^d^ [wt%]
MOPs-I	761	447	0.211	—
MOPs-Pd-I	744	422	0.199	2.5
MOPs-II	664	506	0.225	—
MOPs-Pd-II	623	502	0.225	2.4

a Surface area calculated from the nitrogen adsorption isotherm using the BET method.

b The micropore volume derived using a *t*-plot method based on the Halsey thickness equation.

c Total pore volume at *P*/*P*_0_ = 0.99.

d Data were obtained by inductively coupled plasma mass spectrometry (ICP-AES).

The thermal stability of the MOPs and MOPs-Pd was also assessed by TGA. The TGA traces obtained from MOPs and MOPs-Pd are shown in Fig. 4. The data analysis has been performed, and results are also shown in Fig. 4. These results show that MOPs and MOPs-Pd exhibit good thermal stability in nitrogen. It is obvious to see that the *T*_5%_ and *T*_10%_ of the MOPs-II and MOPs-Pd-II are lower compared with MOPs-I and MOPs-Pd-I. This is because of the large amount CH_2_ in MOPs-I and MOPs-Pd-I.

**Fig. 4:**
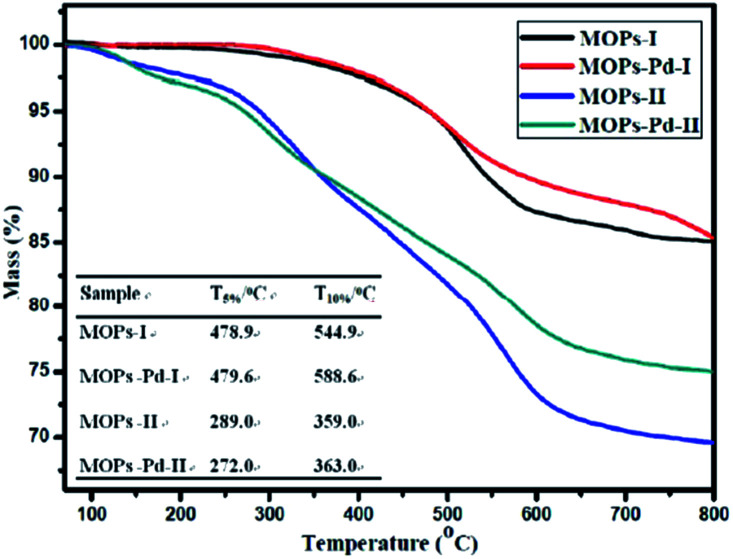
TGA curves of MOPs and MOPs-Pd.

MOPs and MOPs-Pd were subjected to SEM and TEM analyses, and the results are shown in Fig. 5 and 6. We can see a large number of pores in MOPs and MOPs-Pd from the SEM imagines, and uniformly distributed Pd nanoparticles in MOPs-Pd from the TEM images. No remarkable change in terms of the morphology of the materials occurs after loading the palladium species. Then, scanning electron microscopy elemental mapping was employed to investigate the composition of MOPs-Pd. The results are shown in ESI† (Section IV). Obviously, the metal Pd in MOPs-Pd-I and II are distributed in the support with a high degree of dispersion. Meanwhile, C, N, Pd and Cl are observed from these images, implying those are the major elements to construct the MOPs-Pd catalyst.

**Fig. 5:**
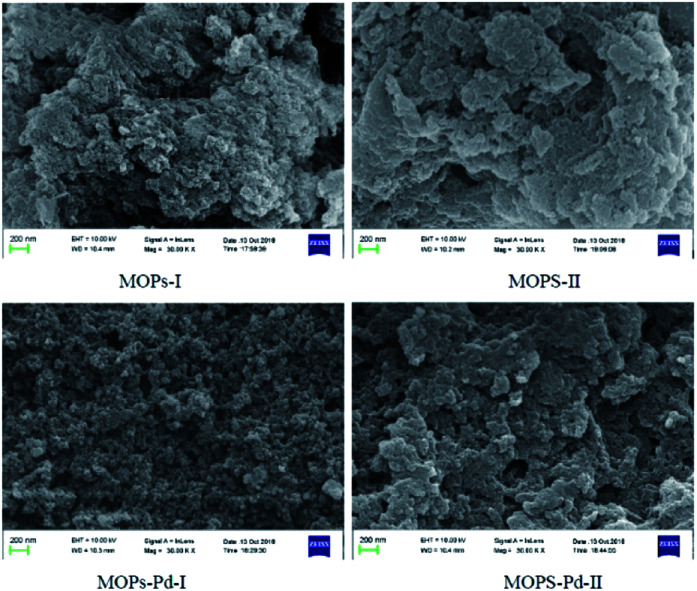
SEM image of MOPs and MOPs-Pd.

**Fig. 6:**
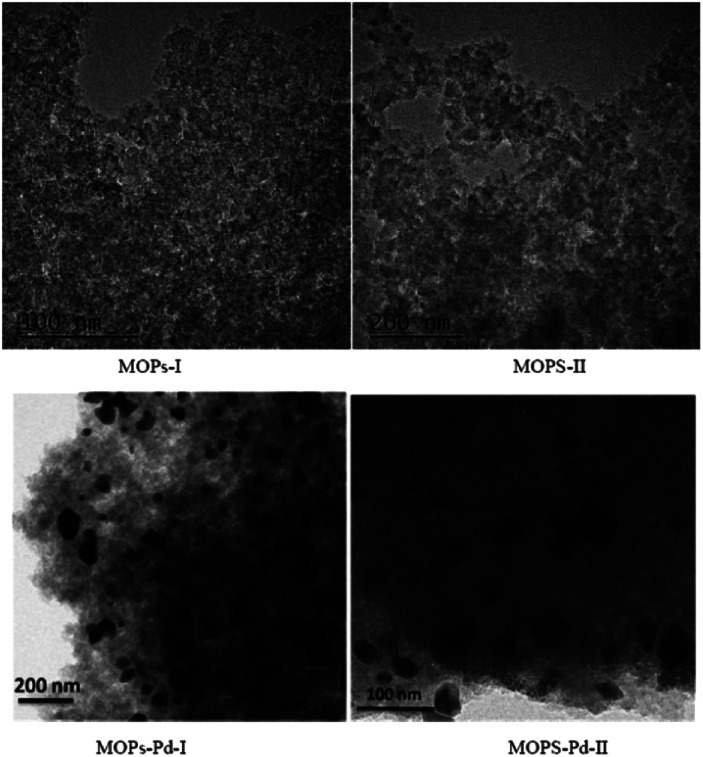
TEM image of MOPs and MOPs-Pd.

Then, we investigated the activities of the MOPs-Pd catalysts to determine the potential relationships between the structure and catalyst activity. To check the catalytic activity of the MOPs-Pd in the Heck coupling reaction, iodobenzene 1a and ethyl acrylate 2a were taken as the model substrate in presence of MOPs-Pd catalyst for optimization of the reaction condition. First, the reaction of 1a with 2a was carried out in the present of Et_3_N with MOPs-Pd-I as catalysis in EtOH under reflex to afford 3a in 75% yield. Then, a series of experiments was carried out to screen the reaction conditions, including catalysis, base, solvent, and reaction temperature. The optimal results were obtained when the reaction of 1a with 2a was carried out in the present of Et_3_N with MOPs-Pd-I as catalysis in DMF at 120 °C for 1.5 h to afford 3a in 96% yield. Under the optimal conditions, we carried out a series of reactions of 1 with 2 aiming to determine its scope. As shown in Table 2, the MOPs-Pd-I catalyzed reaction proved to be suitable for a series of 1 and 2 bearing varied groups, affording the corresponding substituted 3a–o (entry 1–15) in very high yields.

**Table 2:** Heck reaction catalysed by MOPs-Pd-I^a^

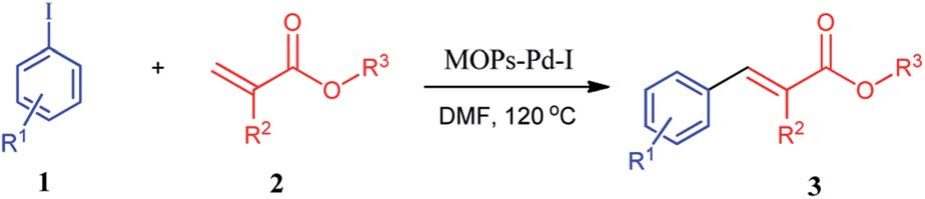					
Entry	R^1^	R^2^	R^3^	3	Yield^b^ (%)
1	H	H	Et	3a	96
2	4-Me	H	Et	3b	98
3	4-MeO	H	Et	3c	97
4	4-Cl	H	Et	3d	95
5	4-NO_2_	H	Et	3e	93
6	4-CN	H	Et	3f	93
7	3-Me	H	Et	3g	94
8	3,5-(Me)_2_	H	Et	3h	97
9	H	H	Me	3i	98
10	3-Me	H	Me	3j	95
11	H	H	Bu	3k	97
12	3-Me	H	Bu	3l	94
13	H	H	H	3m	94
14	4-Me	H	H	3n	95
15	4-MeO	Me	Me	3o	90

a Reaction conditions: 1a (2.5 mmol), 2a (3.7 mmol), Et_3_N (3.7 mmol), MOPs-Pd-I (50 mg, 0.28 mol%), DMF (10 mL), 120 °C, 1.5 h.

b Isolated yields.

To determine the active catalyst, we did two experiments in the same condition as Table 2, entry 1. Those two reactions were quenched after the reaction carried out for 20 minutes. Then, we separated one of the reactions, where the yield of compound 3a is 61%. In another reaction, the heterogeneous catalyst was separated from the reaction mixture by filtering, and then the reaction liquid was allowed to react for another 70 minutes under the same conditions. No significant change in the yield of compound 3a, indicating that the catalyst for the reaction was not the dissolved Pd species leached from the heterogeneous catalyst.

Recovery and recycling of solid heterogeneous catalysts is one of the major concerns in metal-supported catalysis, while supported metal catalyst usually undergo leaching of metal species to solution.^29^ Thus, we investigated the recycling performance in Heck reaction, and the results are shown in Fig. 7. The reaction was conducted in the present of Et_3_N with MOPs-Pd as catalysis in DMF at 120 °C for 1.5 h. Then, the catalyst was recovered by filtering, washing with water and ethyl acetate. Finally, the recovered catalyst was dried in an oven for 2.0 h. After six runs, the reused MOPs-Pd-I and II are still capable of catalyzing the reaction in 93% and 91% yield, respectively. This clearly reveals a slight decrease in catalytic activity and product yield. In addition, the morphology of recovered catalyst was analyzed by the SEM (see ESI,† Section IV), and the results show that there is no remarkable change in terms of the morphology of the materials. The contents of Pd in recovered MOPs-Pd-I and II were 2.3% and 2.1% by ICP-AES, implying a slight leaching of palladium species.

**Fig. 7:**
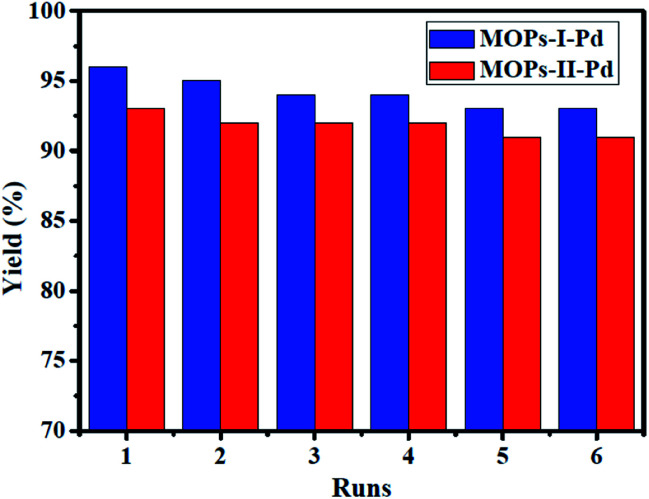
Recycle test of MOPs-Pd in Heck reaction.

To extend the utility of MOPs-Pd in the carbon–carbon coupling reactions, we examined other organic reactions. Suzuki–Miyaura reaction is an important palladium-catalyzed cross coupling in organic synthesis. Therefore, we examined the MOPs-Pd catalysts in Suzuki–Miyaura reaction. First, the reaction of 1a with 4a was put together in the present of K_3_PO_4_ and MOPs-Pd-I in MeOH under reflex. As monitored by TLC, the reaction proceeded smoothly and the yield of 5a reached 91%. Then we investigated the optimization of the reaction conditions, including catalysis, base, solvent, and reaction temperature. A series of experiments revealed that EtOH/H_2_O (*V*_EtOH_/*V*_H_2_O_ = 2 : 1) was effective for the synthesis of 5a. The yield of 5a reached 97% when the reaction of 1a with 4a was performed in the present of K_3_PO_4_ with MOPs-Pd-I as a catalyst at 80 °C for 1.0 hour. In this reaction, the MOPs-Pd-I catalyst also can be reused for 5 times with no significant decrease in activity and the Pd content of the recovered catalyst is 2.36% by ICP-AES.

Under the optimal conditions, we carried out a series of reactions of 1 with 4 aiming to determine its scope. In Table 3, a range of halogenated benzene 1 and aryl boronic acid 4 with electron-donating group and electron-withdrawing group were applied to the conditions in parallel, affording the corresponding substituted biphenyl 5b–i in high yields. It is worth noting that the cross-coupling reactions can proceed smoothly when aryl bromide was used in the reaction (entry 12).

**Table 3:** Suzuki–Miyaura reaction catalysed by MOPs-Pd-I^a^

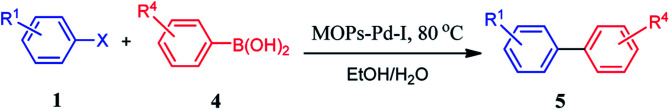					
Entry	R^1^	X	R^4^	5	Yield^b^ (%)
1	H	I	H	5a	97
2	H	I	2-Me	5b	96
3	H	I	3-Me	5c	99
4	H	I	4-Me	5d	98
5	H	I	2 F	5e	96
6	H	I	3 F	5f	95
7	H	I	4 F	5g	97
8	H	I	4-CN	5h	95
9	4-Me	I	H	5d	98
10	4-OMe	I	H	5i	99
11	4-CN	I	H	5h	94
12^c^	H	Br	H	5a	92

a Reaction conditions: 1a (2.5 mmol), 4a (3.0 mmol), K_3_PO_4_ (5.0 mmol), MOPs-Pd-I (50 mg, 0.28 mol%), EtOH/H_2_O (10 mL), 80 °C, 1.0 h.

b Isolated yields.

c The reaction time was 3.0 h.

In summary, a simple and low-cost method for synthesis of palladium complexes supported on microporous organic polymers was described. The MOPs-Pd catalysts were constructed based on highly stable microporous material, and characterized by FT-IR, TGA, SEM, TEM, N_2_ sorption, XPS and ICP. These heterogeneous catalysts displayed outstanding catalytic activities in Heck reaction and Suzuki coupling reaction. In these reactions, the MOPs-Pd catalyst was easily recovered and reused without loss of catalytic activity. The potential utilization and application of these heterogeneous catalysts are currently under investigation in our laboratory.

## Conflicts of interest

The authors declare that there are no conflicts of interests.

## Supplementary Material

RA-009-C9RA07303E-s001
