# Supplementary material for: Palladium catalyst immobilized on functionalized microporous organic polymers for C–C coupling reactions
Source: RSC Adv. 2019 Oct 28;9(59):34595–600. doi: 10.1039/c9ra07303e (PMC9073918; doi:10.1039/c9ra07303e)

Electronic Supplementary Material (ESI) for RSC Advances.
This journal is © The Royal Society of Chemistry 2019

Electronic Supplementary Information

Palladium catalyst immobilized on functionalized microporous organic polymers for C-C coupling reactions

Wei Xu,‡ Cijie Liu,‡ DexuanXiang,* Qionglin Luo, You Shu, Hongwei Lin, Yangjian Hu, Zaixing Zhang, and Yuejun Ouyang*

Hunan Engineering Laboratory for Preparation Technology of Polyvinyl Alcohol (PVA) Fiber Material, Institute of Organic Synthesis, Huaihua University, Huaihua 418000, China. E-mail: dexuanxiang@126.com

‡ Wei Xu and Cijie Liu contributed equally to this work.

Table of contents 1

I. General 2

II. Synthesis of **MOPs** 2-3

III. Synthesis of **MOPs-Pd** 3

IV. Analytical data of **MOPs** and **MOPs-Pd** 4-7

V. Synthesis and analytical data of **3** and **5** 7-32

I.General

All reagents were purchased from commercial sources and used without treatment, unless otherwise indicated. FT-IR spectra were recorded under ambient conditions in the wavenumber range of 4000-400 cm^{-1} using an FT-IR Bruker (EQUINOX 55) spectrometer. Nitrogen adsorption-desorption was assessed using a Surface and Pore Size Analysis Instrument (3H-2000PM1, Beishide Instrument-S&T Co., Ltd., China) at 77.3K. All samples were out gassed under vacuum at 80°C for 5 h prior to the measurement. The special surface area was calculated by the BET method. The micropore volume derived using a t-plot method based on the Halsey thickness equation. The pore size distribution was obtained by applying the BJH formalism to both the adsorption and desorption branch of the isotherm. XPS was performed on a thermo ESCALAB 250XI by using Al Ka radiation as excitation source. TGA was performed on a thermogravimetric analyser (DSC Q2000) under N_2 environment with a temperature rate of 10°C/min from room temperature to 800°C. The structure and morphology of the MOPs and MOPs-Pd were observed using transmission electron microscopy (TEM, FEI Tecnai G20) and scanning electron microscopy (SEM, zeiss, sigma HD). The content of Pd was measured by inductively coupled plasma emission spectrometry (ICP-AES, Agilent, Icpoes 730). The products were purified by recrystallization and column chromatography over silica gel. ^{1}H NMR and ^{13}C NMR spectra were recorded at 25 °C at 500 MHz and 125 MHz, respectively, with TMS as internal standard.

II. Synthesis of MOPs

Synthesis of MOPs-I: $FeCl_3$ (anhydrous, 0.09mol) was added to a solution of benzene (0.06mol), 1,10-Phenanthroline hydrochloride (0.03mol), and formaldehyde dimethyl acetal (FDA, 0.12mol) in 50mL 1,2-dichloroethane (DCE). The resulting mixture was stirred at room temperature for good mixing, and then stirred at 45°C for 5h to form original network, and then heated at 80°C for 48h to react completely. The resulting precipitate was washed with methanol (20mL) and then washed with dilute hydrochloric acid for 3 times (3 ×40mL). The solid reacted with KOH aqueous solution at 50°C for 40 minutes, washed with methanol for 3 times (3 ×20mL), then washed with methanol in a Soxhlet for 24 h, and finally dried under reduced pressure at 60°C for 24h to give MOPs-I.

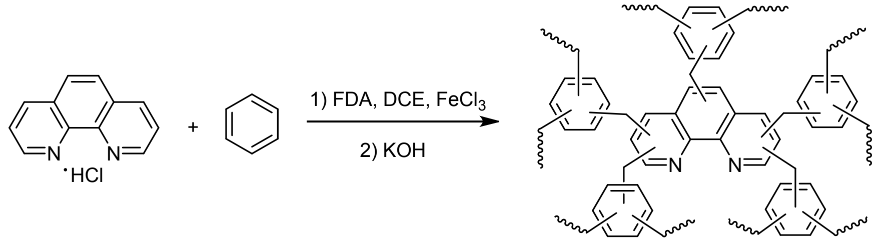

Synthesis of MOPs-II: $AlCl_3$ (anhydrous, 0.25mol) was added to a solution of benzene (0.03mol) and 1,10-Phenanthroline hydrochloride (0.025mol) in 80mL chloroform. The resulting mixture was stirred at 0°C for good mixing, and then stirred at 50°C for 2h to form original network, and then heated at 80°C for 40h to react completely. The resulting precipitate was washed with methanol (20mL) and then washed with dilute hydrochloric acid for 3 times (3 ×40mL). The solid reacted with KOH aqueous solution at 50°C for 40 minutes, washed with methanol for 3 times (3 ×20mL), then washed with methanol in a Soxhlet for 24 h, and finally dried under reduced pressure at 60°C for 24h to give MOPs-II.

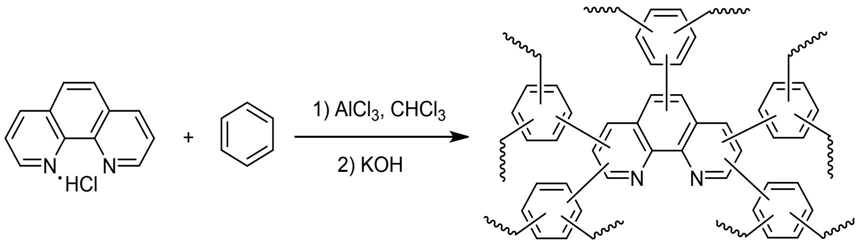

III. Synthesis of MOPs-Pd

Synthesis of MOPs-Pd-I: MOPs-I (2.0g), $PdCl_2$ (0.20g) and 50mL of anhydrous tetrahydrofuran were placed in a three-necked flask. Then, the reaction mixture was stirred under refluxing for 24h. Finally, the solid was filtered and washed with methanol 3 times (3 ×20mL), then washed with acetone in a Soxhlet for 40h, and dried under reduced pressure at 60°C for 24h to give MOPs-Pd-I.

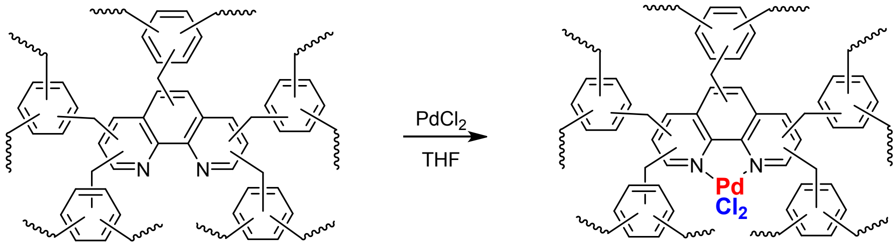

Synthesis of MOPs-Pd-II: MOPs-II (2.0g), $PdCl_2$ (0.20g) and 50mL of anhydrous tetrahydrofuran were placed in a three-necked flask. Then, the reaction mixture was stirred under refluxing for 24h. Finally, the solid was filtered and washed with methanol 3 times (3 ×20mL), then washed with acetone in a Soxhlet for 40h, and dried under reduced pressure at 60°C for 24h to give MOPs-Pd-II.

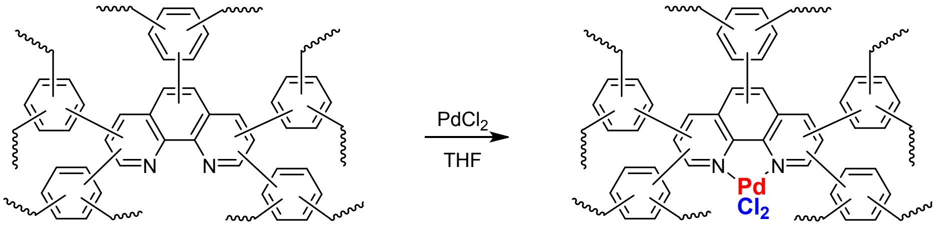

IV. Analytical data of MOPs and MOPs-Pd

1) SEM elemental mapping imagine of MOPs-Pd-I

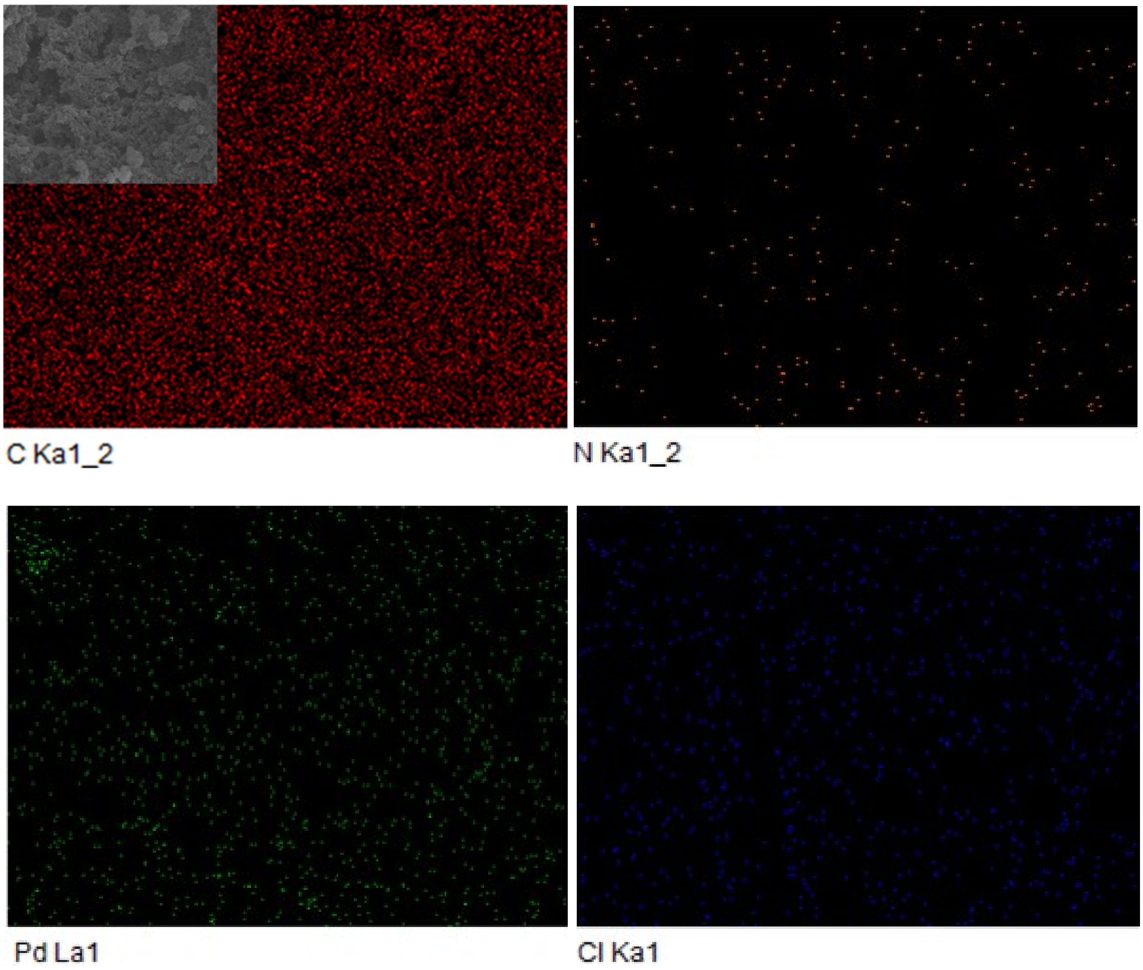

MOPs-Pd-I

2) SEM elemental mapping imagine of MOPs-Pd-II

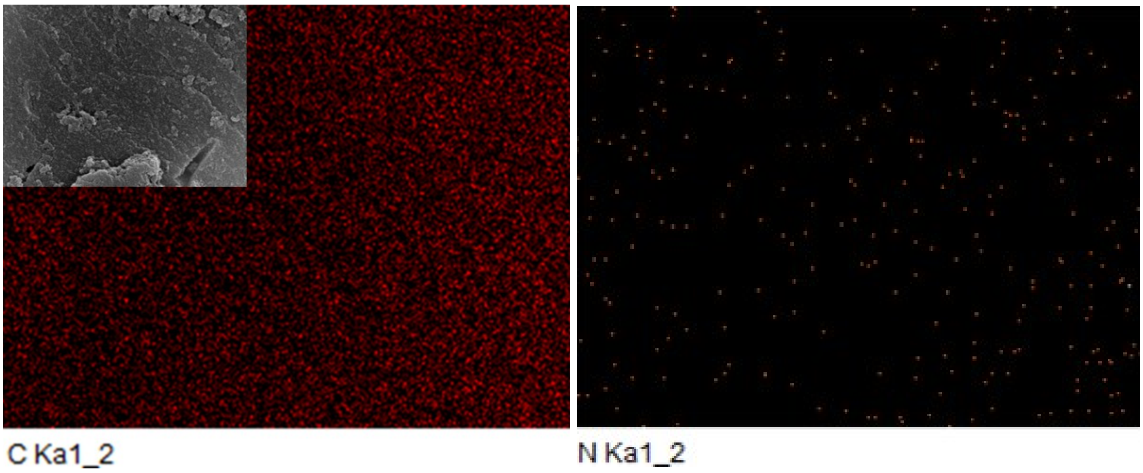

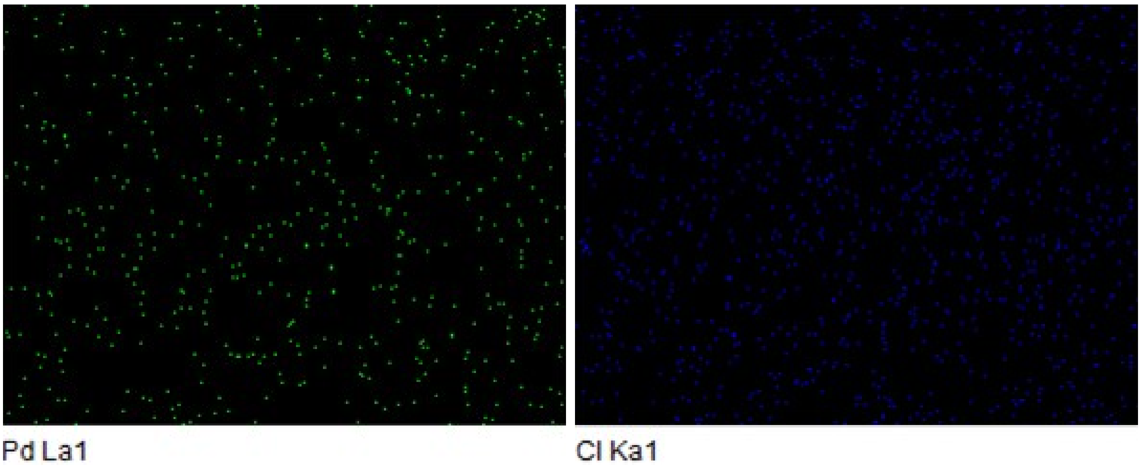

MOPs-Pd-II

3) TEM imagine of recycled MOPs-Pd

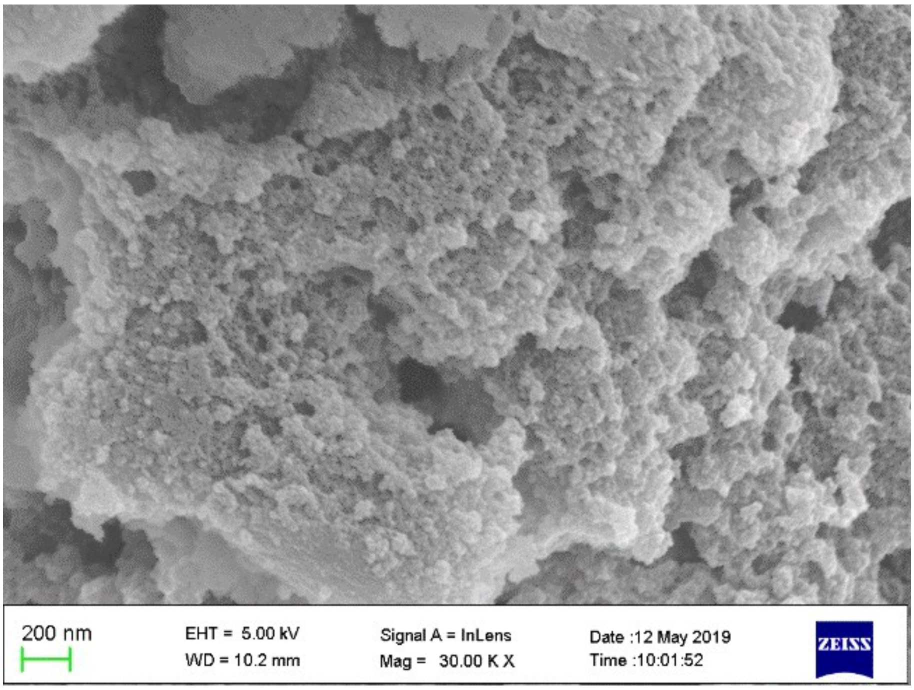

TEM imagine of recycled MOPs-Pd-I

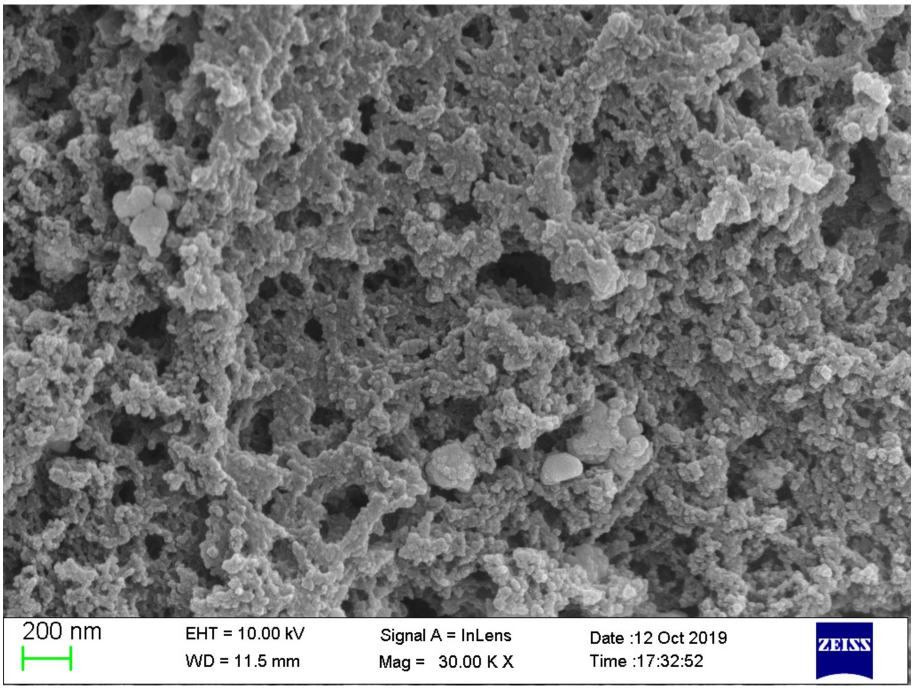

TEM imagine of recycled MOPs-Pd-II

4) SEM elemental mapping imagine of MOPs-Pd-II

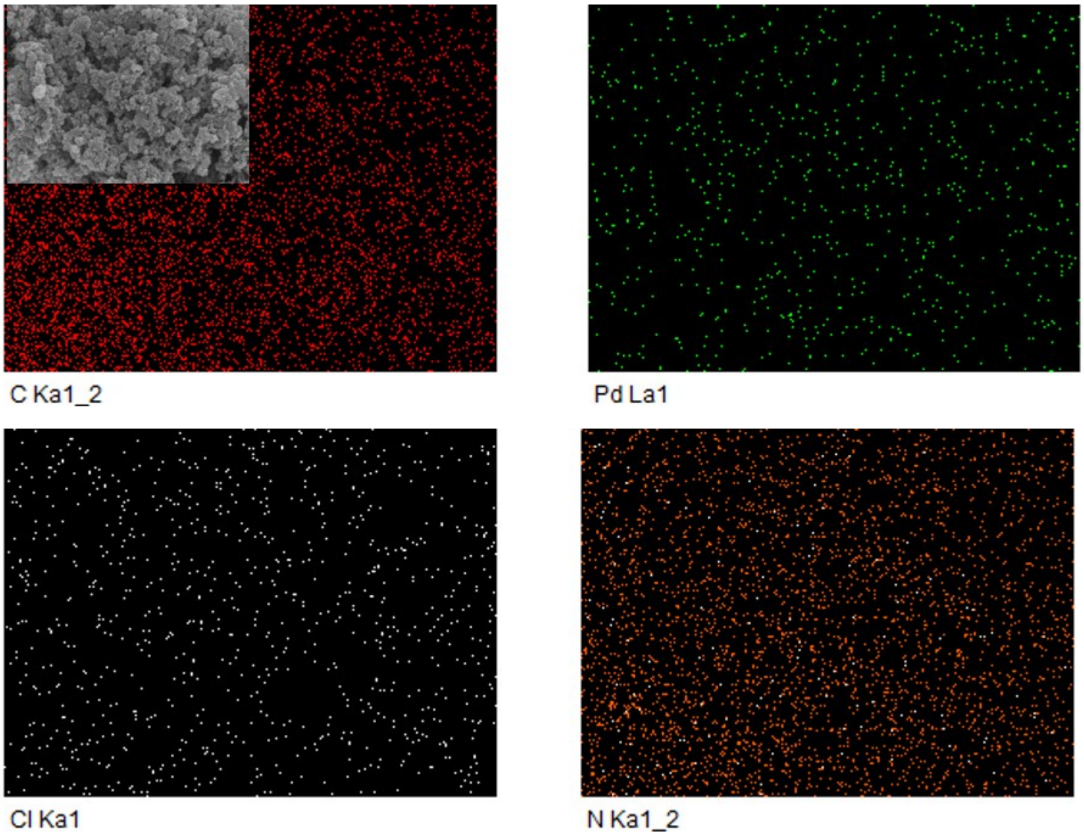

Recycled MOPs-Pd-I

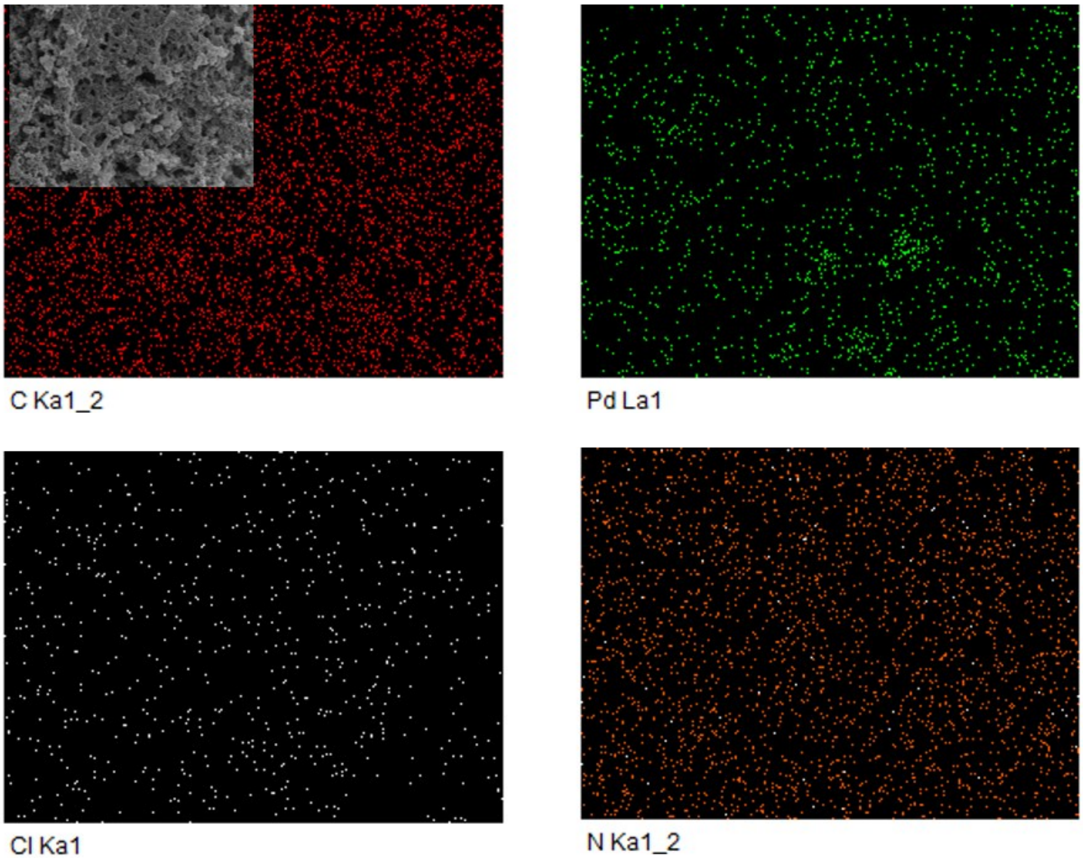

Recycled MOPs-Pd-II

V. Synthesis and analytical data of 3 and 5

1) Typical procedure for the preparation of 3 (3a as example):

MOPs-Pd-I(50mg) was added to a solution of iodobenzene **1a** (2.5mmol), ethyl acrylate **2a** (3.7mmol), and Et_3N (3.7mmol) in 10mL DMF. The mixture was stirred under a N_2 atmosphere at 120°C for 1.5 hour. After the substrate iodobenzene **1a** was consumed as indicated by TLC, the mixture was poured into ice-water (100mL) under stirring. The mixture was extracted with dichloromethane (3 × 20mL), and the combined organic phase was washed with water (3× 20mL), dried over $MgSO_4$, filtered and concentrated in vacuo. The crude product was purified by flash chromatography (silica gel, petroleum ether: diethyl ether = 20: 1) to give **3a** as a white solid (422 mg, 96%).

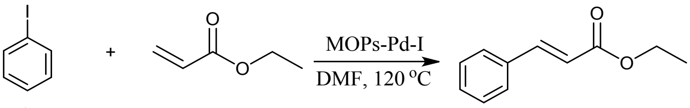

1a **2a** **3a**

2) Analytical data and Copies of NMR spectra for 3

Compound **3a:** ^{1}H NMR (400 MHz, $CDCl_3$) δ 7.69 (d, J = 16.0 Hz, 1H), 7.54 – 7.48 (m, 2H), 7.40 – 7.33 (m, 3H), 6.43 (d, J = 16.0 Hz, 1H), 4.26 (q, J = 7.1 Hz, 2H), 1.33 (t, J = 7.1 Hz, 3H); ^{13}C NMR (101 MHz, $CDCl_3$) δ 166.94, 144.55, 134.48, 130.18, 128.85, 128.02, 118.30, 60.45, 14.30; IR (KBr) 2986, 2901, 1701, 1638, 1311, 1269, 1175, 1040, 980, 768, 685.

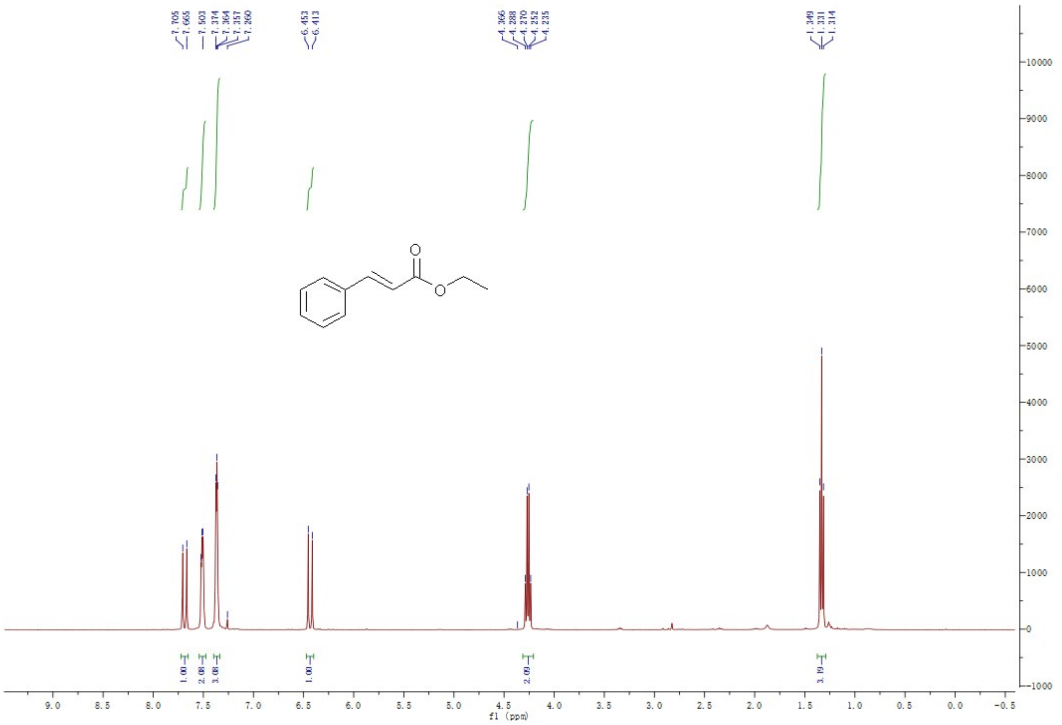

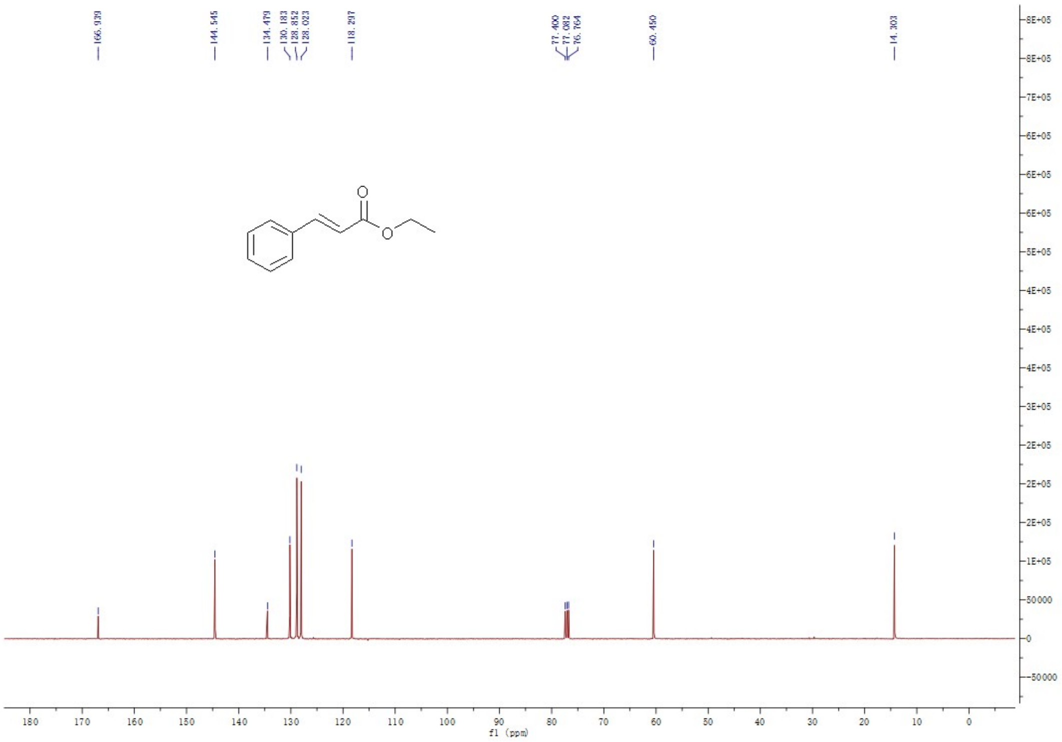

Compound **3b:** ^{1}H NMR (500 MHz, $CDCl_3$) δ 7.66 (d, *J* = 16.0 Hz, 1H), 7.42 (d, *J* = 8.0 Hz, 2H), 7.19 (d, *J* = 7.9 Hz, 2H), 6.39 (d, *J* = 16.0 Hz, 1H), 4.26 (q, *J* = 7.2 Hz, 2H), 2.37 (s, 3H), 1.33 (t, *J* = 7.1 Hz, 3H); ^{13}C NMR (126 MHz, $CDCl_3$) δ 167.22, 144.59, 140.62, 131.72, 129.60, 128.04, 117.16, 60.41, 21.45, 14.34 ; IR (KBr) 2981, 1711, 1636, 1310, 1259, 1165, 1039, 984, 812

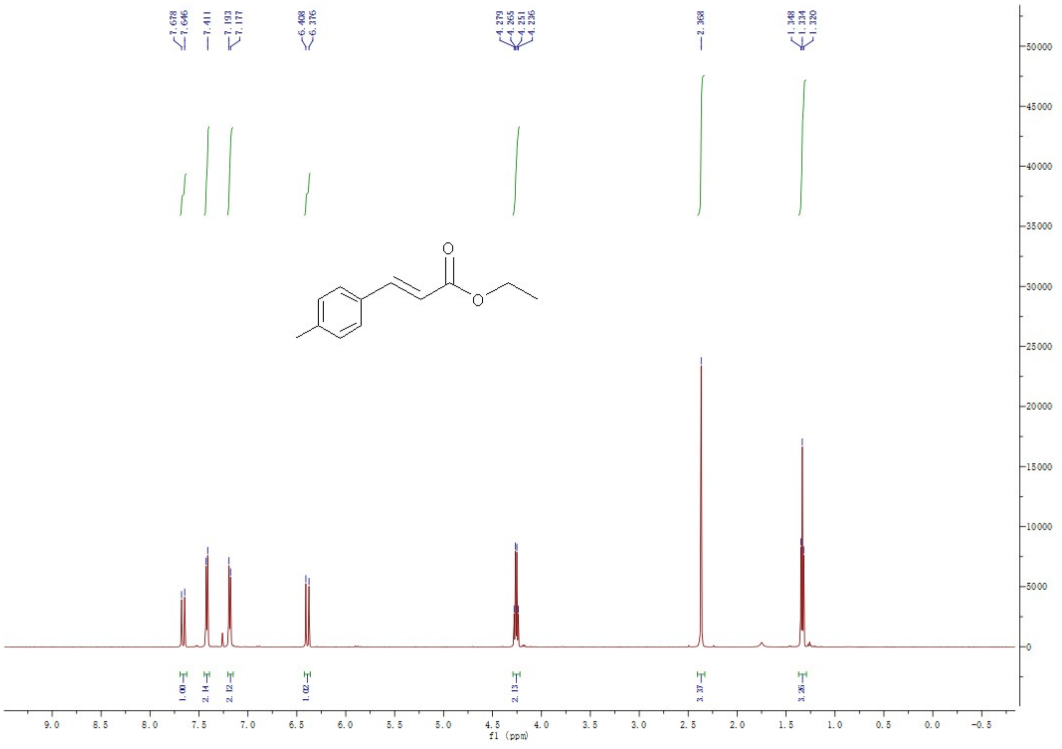

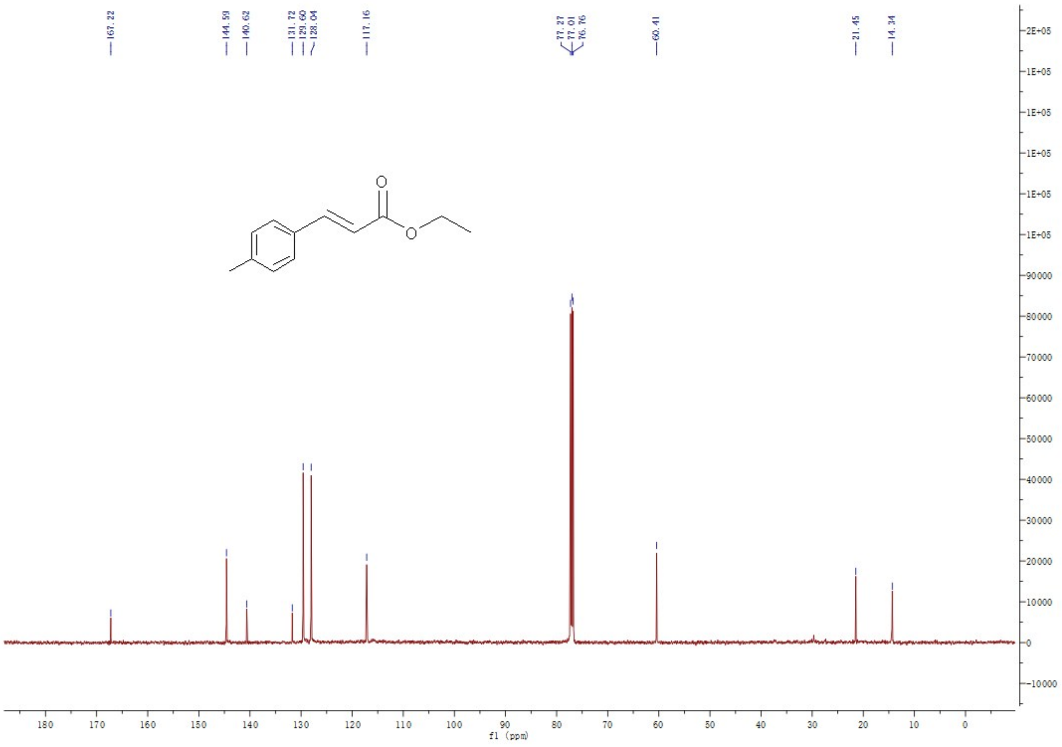

Compound **3c:** ^{1}H NMR (500 MHz, $CDCl_3$) δ 7.63 (d, J = 16.0 Hz, 1H), 7.46 (d, J = 8.7 Hz, 2H), 6.89 (d, J = 8.7 Hz, 2H), 6.30 (d, J = 16.0 Hz, 1H), 4.24 (q, J = 7.1 Hz, 2H), 3.81 (s, 3H), 1.32 (t, J = 7.1 Hz, 3H); ^{13}C NMR (126 MHz, $CDCl_3$) δ 167.35, 161.32, 144.24, 129.68, 127.19, 115.73, 114.30, 60.32, 55.35, 14.35; IR (KBr) 2979, 1707, 1634, 1604, 1512, 1251, 1164, 1031, 983, 828.

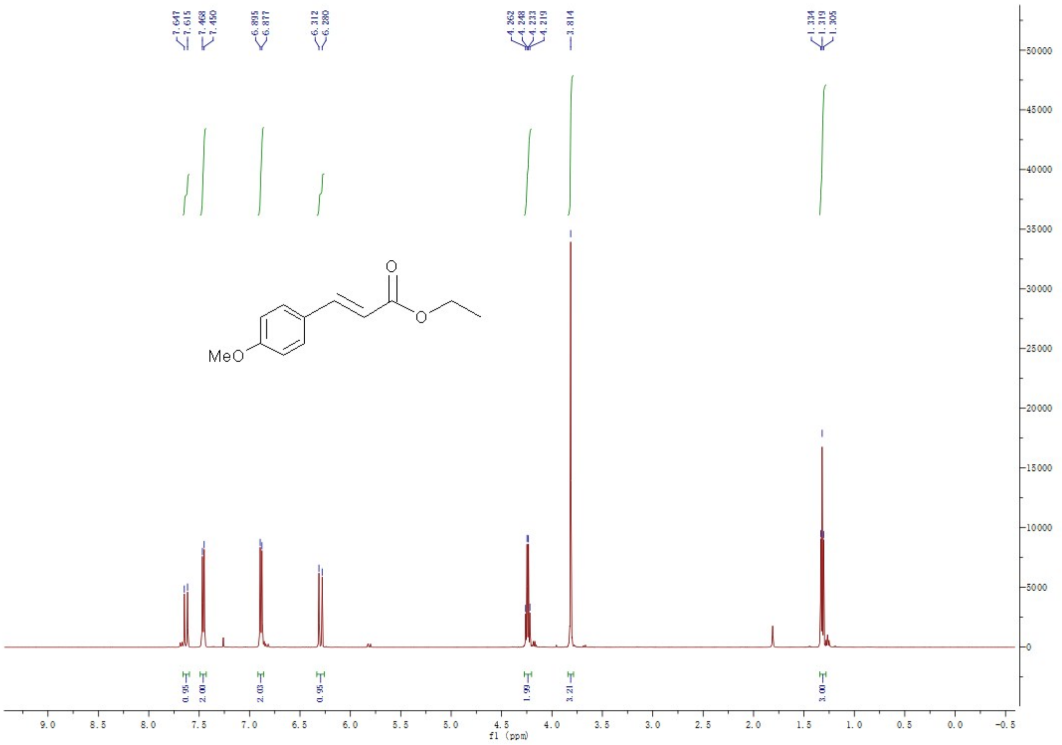

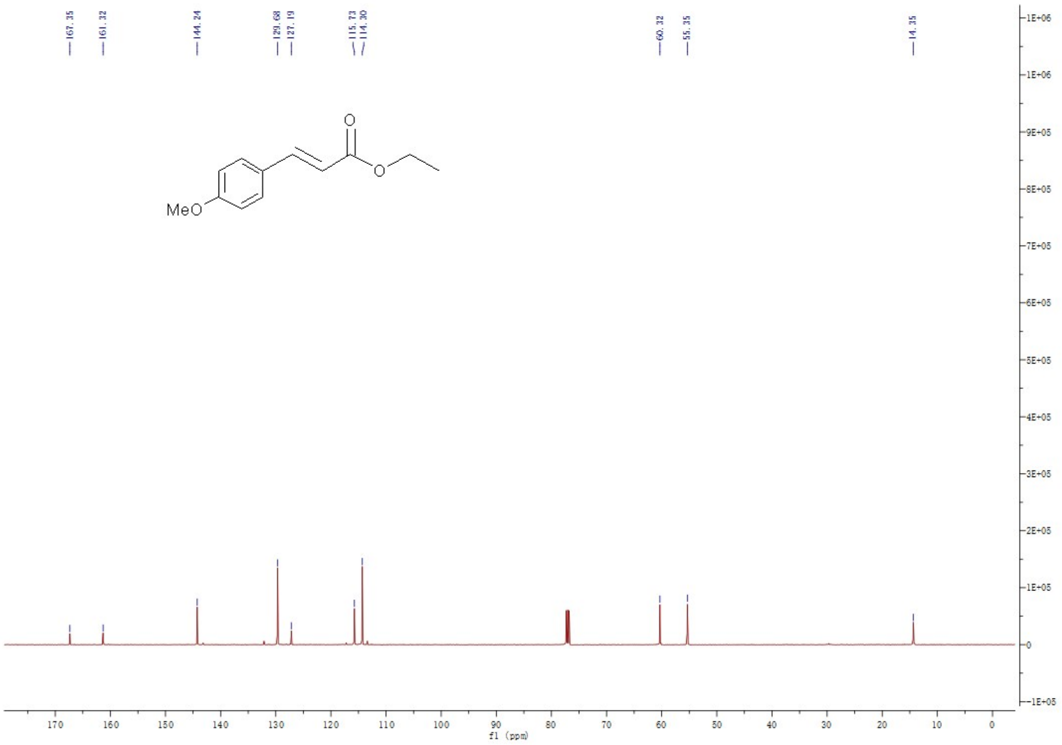

Compound **3d:** ^{1}H NMR (500 MHz, $CDCl_3$) δ 7.61 (d, J = 16.0 Hz, 1H), 7.43 (d, J = 8.5 Hz, 2H), 7.34 (d, J = 8.5 Hz, 2H), 6.39 (d, J = 16.0 Hz, 1H), 4.25 (q, J = 7.1 Hz, 2H), 1.32 (t, J = 7.1 Hz, 3H); ^{13}C NMR (126 MHz, $CDCl_3$) δ 166.74, 143.12, 136.10, 132.94, 129.17, 118.85, 60.62, 14.30; IR (KBr) 2982, 1712, 1639, 1539, 1491, 1310, 1270, 1173, 1089, 1036, 981, 822.

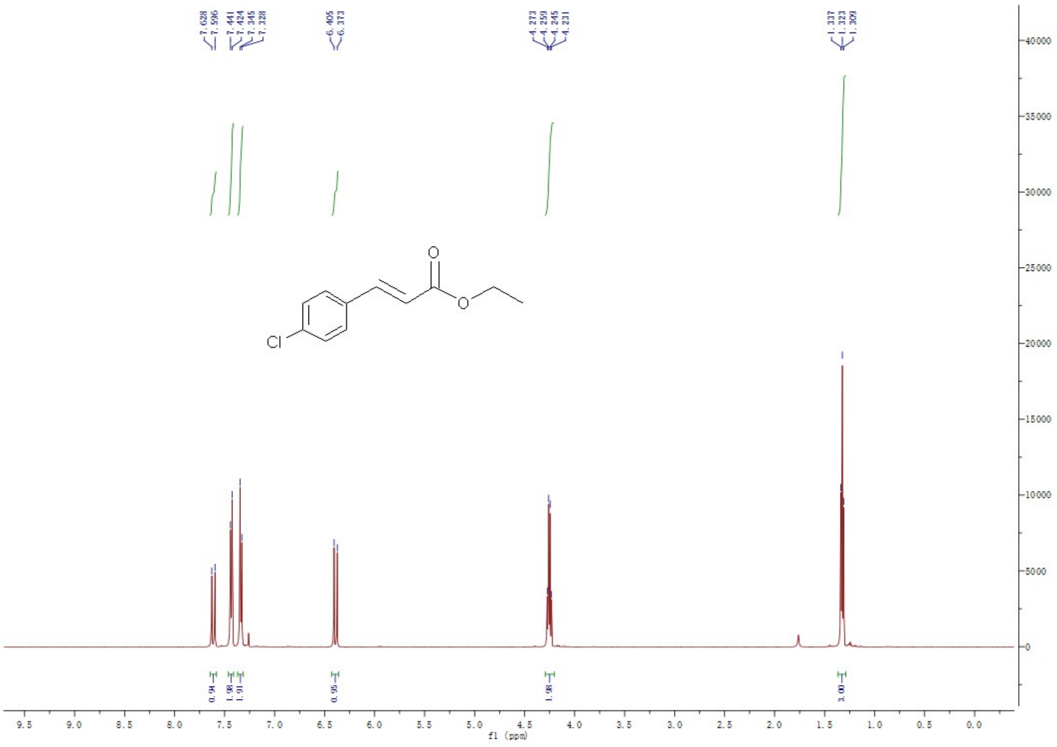

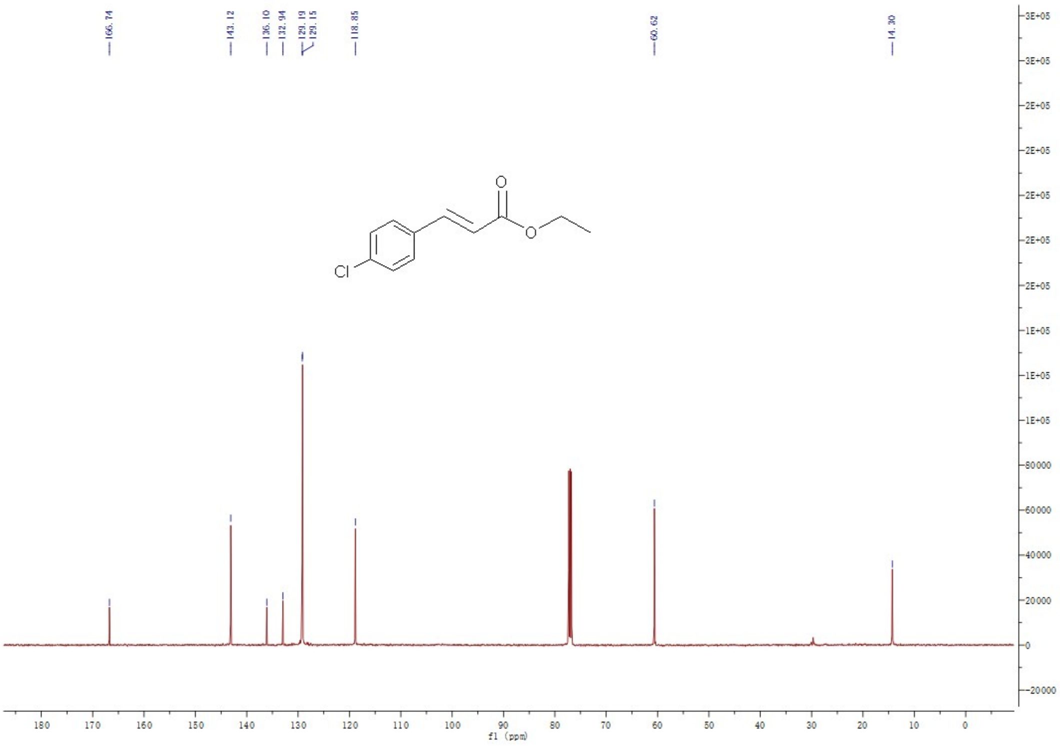

Compound **3e:** ^{1}H NMR (400 MHz, DMSO) δ 8.24 (d, J = 8.7 Hz, 1H), 8.01 (d, J = 8.7 Hz, 1H), 7.75 (d, J = 16.1 Hz, 1H), 6.84 (d, J = 16.1 Hz, 0H), 4.24 (q, J = 7.1 Hz, 1H), 1.30 (t, J = 7.1 Hz, 2H); ^{13}C NMR (101 MHz, DMSO) δ 166.09, 148.49, 142.19, 140.91, 129.85, 129.10, 124.63, 124.33, 122.90, 60.84, 14.56; IR (KBr) 1716, 1578, 1437, 1365, 1176, 1029, 978, 845, 650.

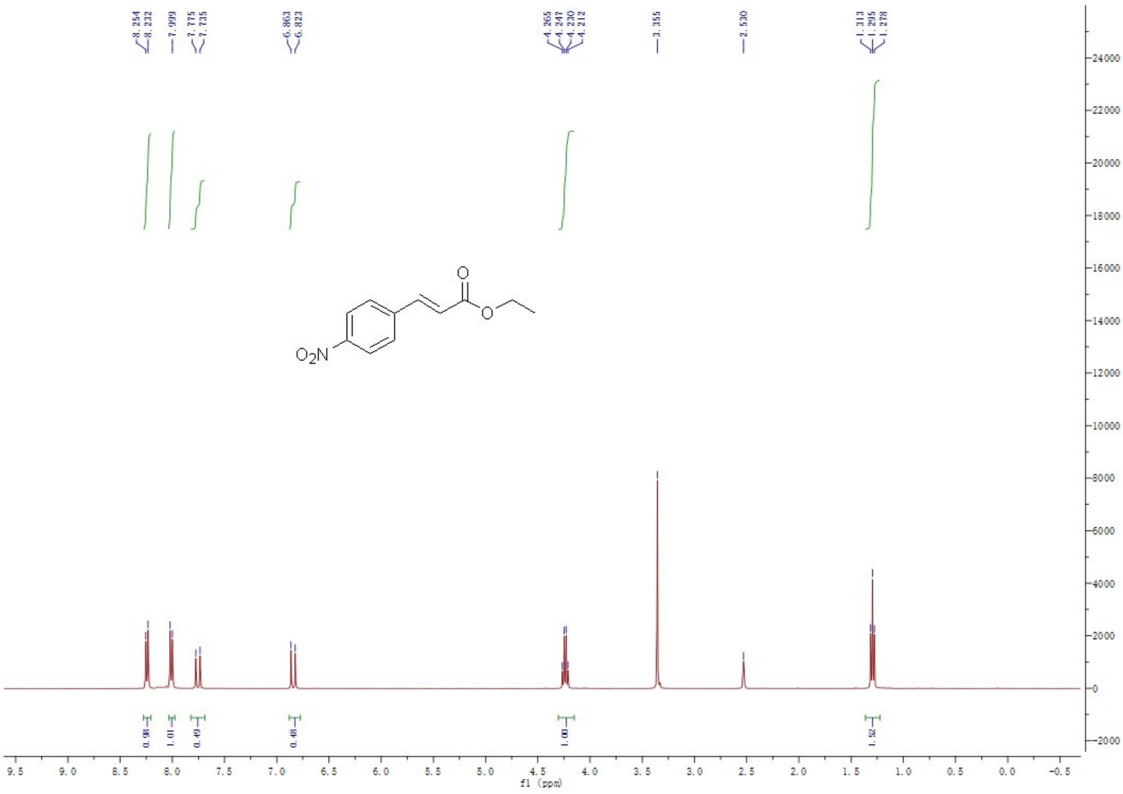

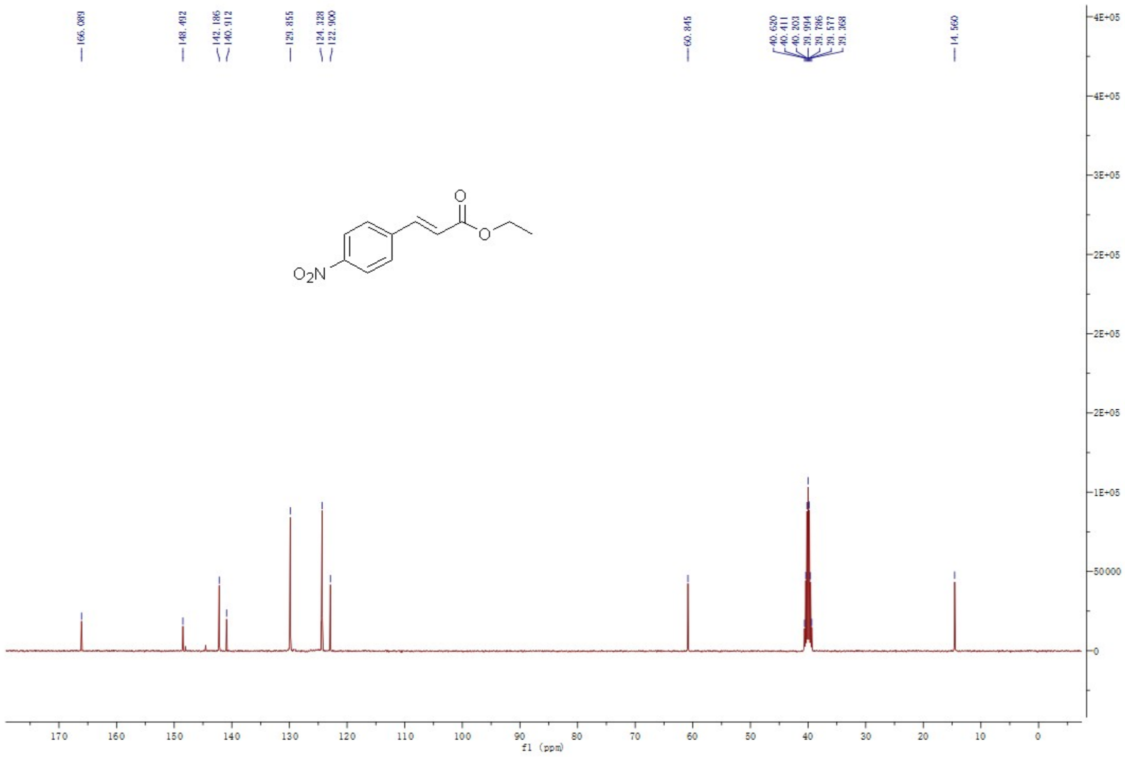

Compound **3f:** ^{1}H NMR (500 MHz, $CDCl_3$) δ 7.64 (t, J = 11.5 Hz, 3H), 7.59 (d, J = 8.2 Hz, 2H), 6.49 (d, J = 16.0 Hz, 1H), 4.26 (q, J = 7.1 Hz, 2H), 1.32 (t, J = 7.1 Hz, 3H); ^{13}C NMR (126 MHz, $CDCl_3$) δ 166.13, 142.12, 138.74, 132.63, 128.37, 121.86, 118.36, 113.32, 60.93, 14.25; IR (KBr) 2987, 1707, 1638, 1310, 1208, 1178, 1001, 849, 826.

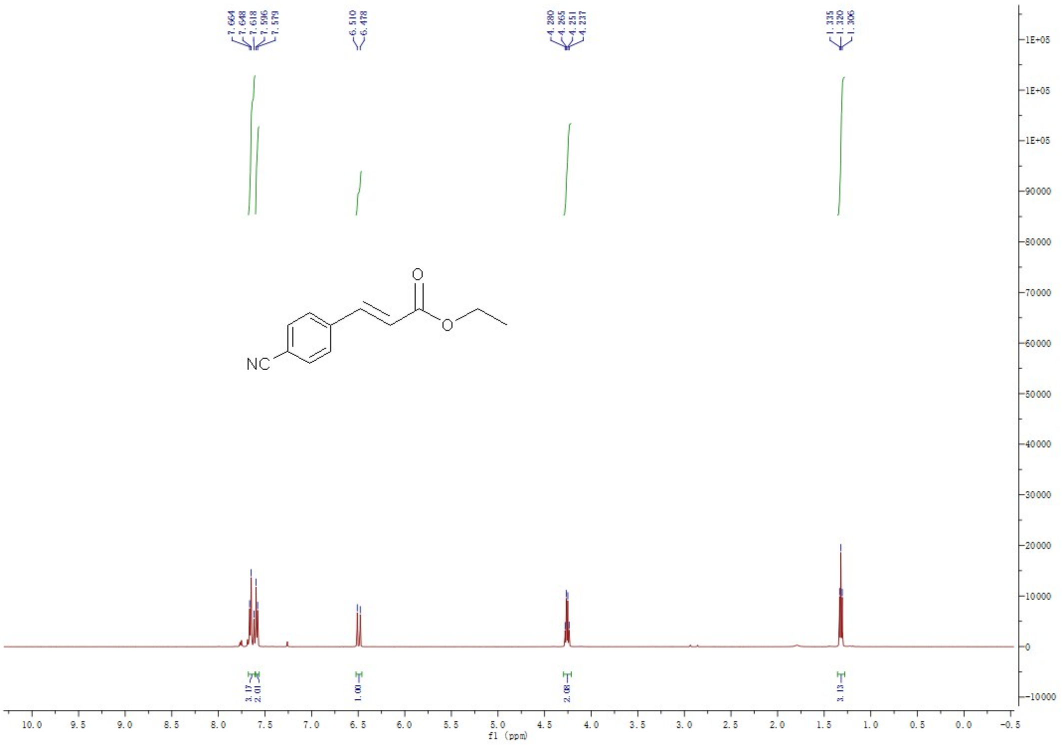

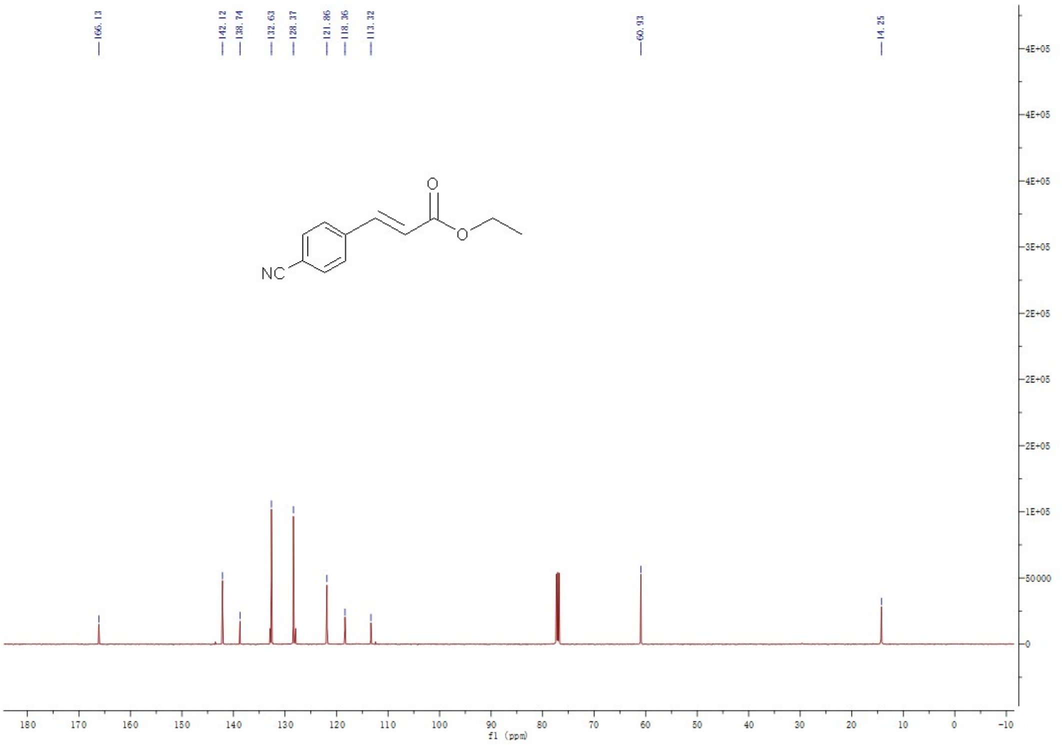

Compound **3g:** ^{1}H NMR (500 MHz, $CDCl_3$) δ 7.71 (d, J = 16.0 Hz, 1H), 7.38 (d, J = 7.3 Hz, 2H), 7.32 (t, J = 7.6 Hz, 1H), 7.24 (d, J = 7.4 Hz, 1H), 6.47 (d, J = 16.0 Hz, 1H), 4.35 – 4.26 (m, 2H), 2.40 (d, J = 17.5 Hz, 3H), 1.43 – 1.34 (m, 3H); ^{13}C NMR (126 MHz, $CDCl_3$) δ 167.10, 144.77, 138.52, 134.41, 131.05, 128.75, 128.71, 125.23, 118.03, 60.46, 21.32, 14.32; IR (KBr) 2980, 1710, 1638, 1310, 1237, 1176, 1162, 1039, 983, 787, 681.

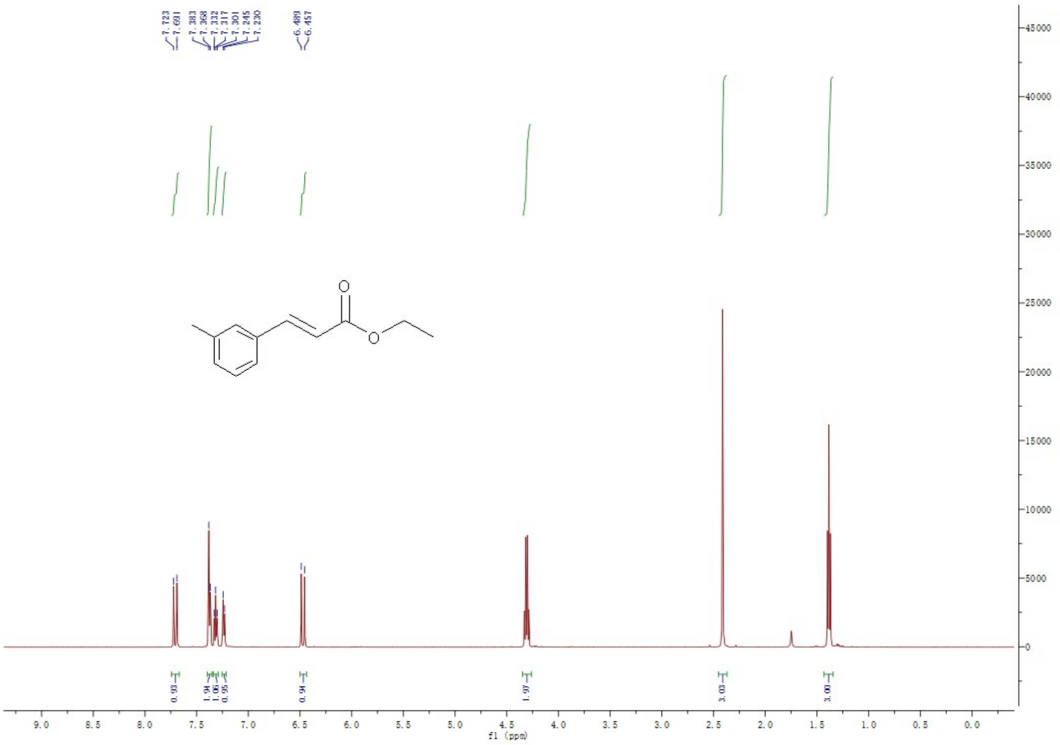

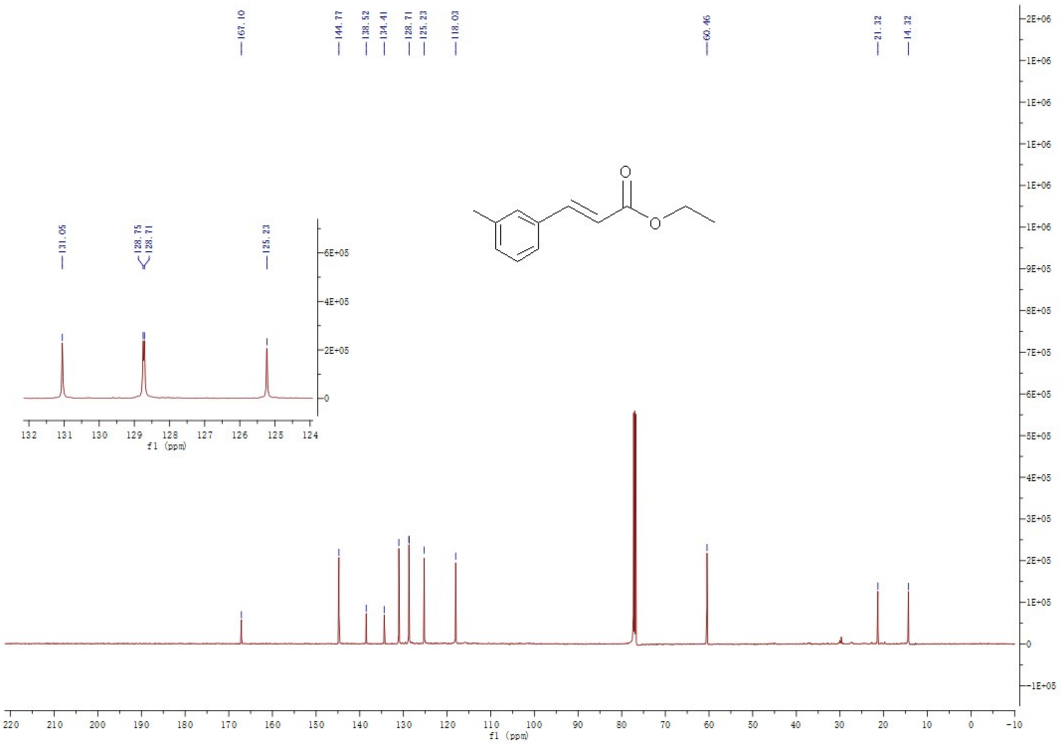

Compound **3h:** ^{1}H NMR (500 MHz, $CDCl_3$) δ 7.63 (d, J = 16.0 Hz, 1H), 7.14 (s, 2H), 7.02 (s, 1H), 6.41 (d, J = 16.0 Hz, 1H), 4.26 (q, J = 7.1 Hz, 2H), 2.33 (s, 6H), 1.34 (t, J = 7.2 Hz, 3H); ^{13}C NMR (126 MHz, $CDCl_3$) δ 167.15, 144.93, 138.38, 134.37, 132.02, 125.94, 117.80, 60.40, 21.18, 14.32; IR (KBr) 2980, 1712, 1637, 1326, 1254, 1177, 1163, 1041, 982, 844, 678.

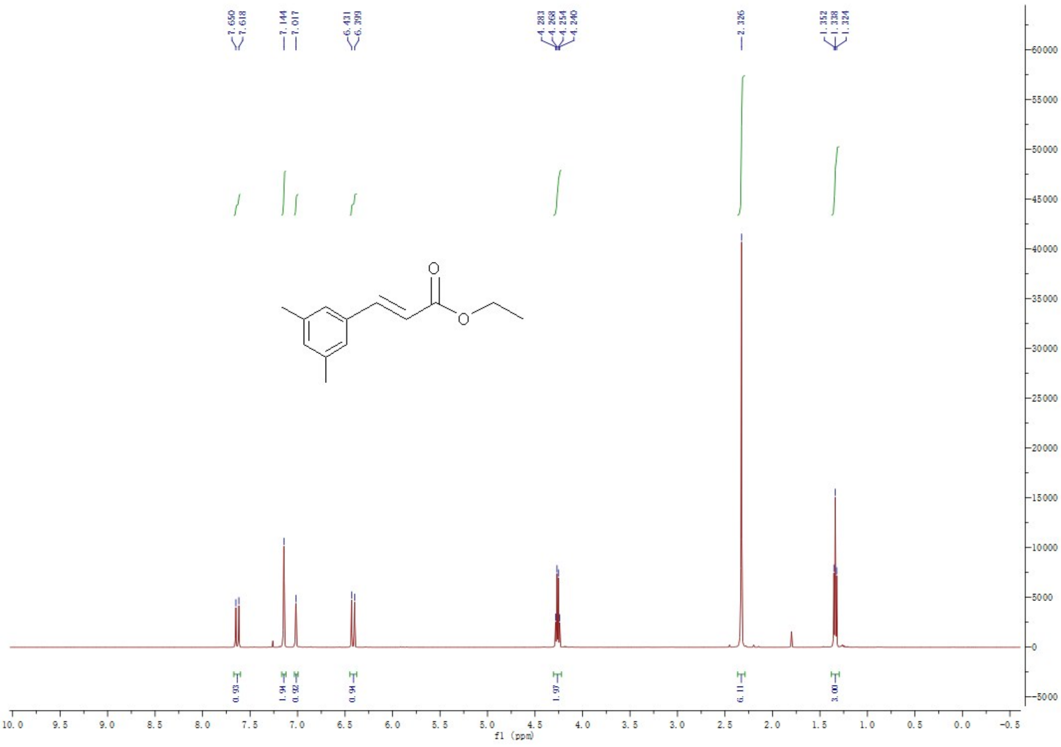

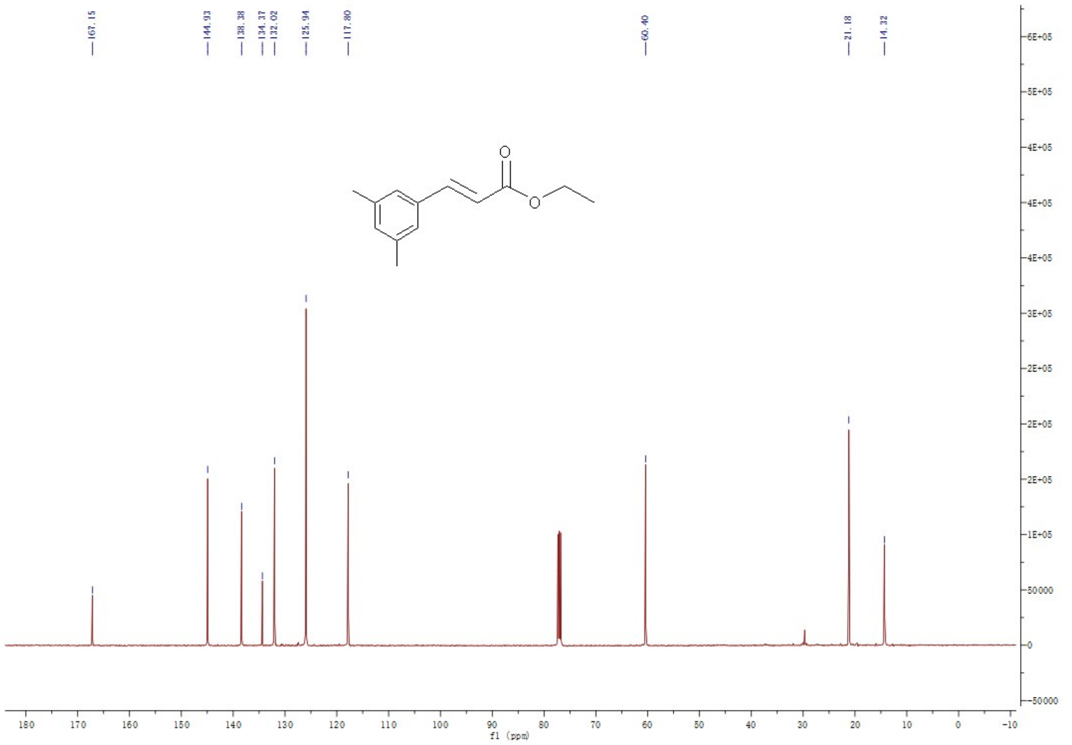

Compound **3i:** ^{1}H NMR (500 MHz, $CDCl_3$) δ 7.69 (d, J = 16.0 Hz, 1H), 7.53 – 7.47 (m, 2H), 7.40 – 7.33 (m, 3H), 6.44 (d, J = 16.0 Hz, 1H), 3.79 (s, 3H); ^{13}C NMR (126 MHz, $CDCl_3$) δ 167.43, 144.89, 134.40, 130.31, 128.91, 128.09, 117.82, 51.70; IR (KBr) 2950, 1716, 1637, 1314, 1275, 1203, 1170, 980, 768, 684.

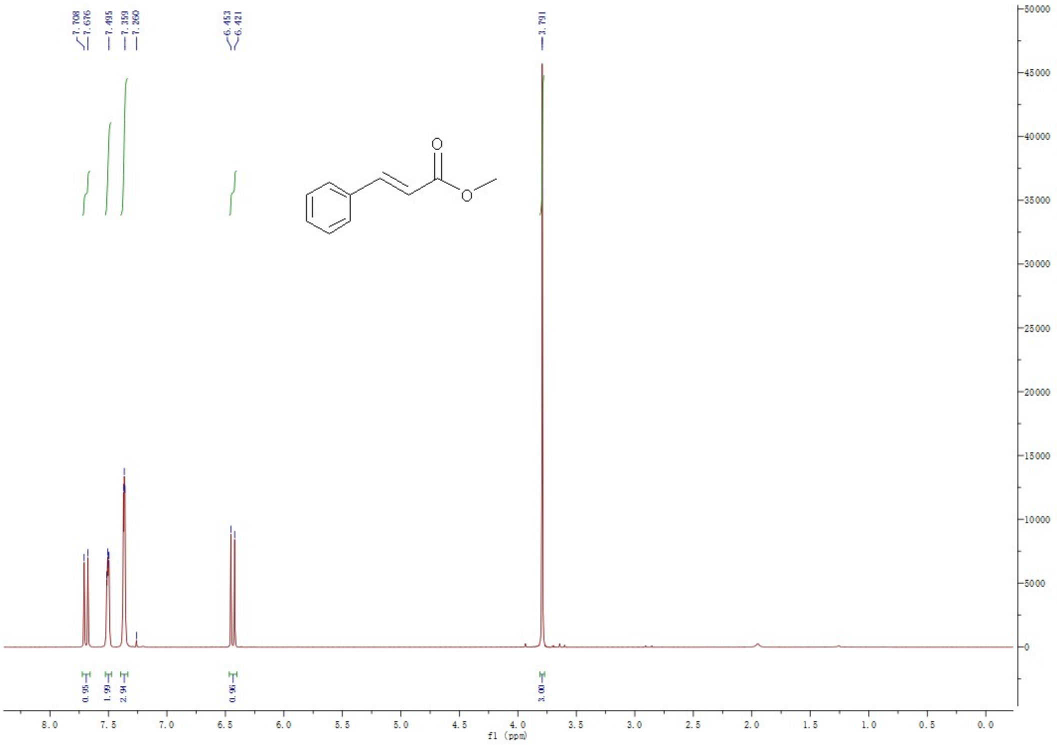

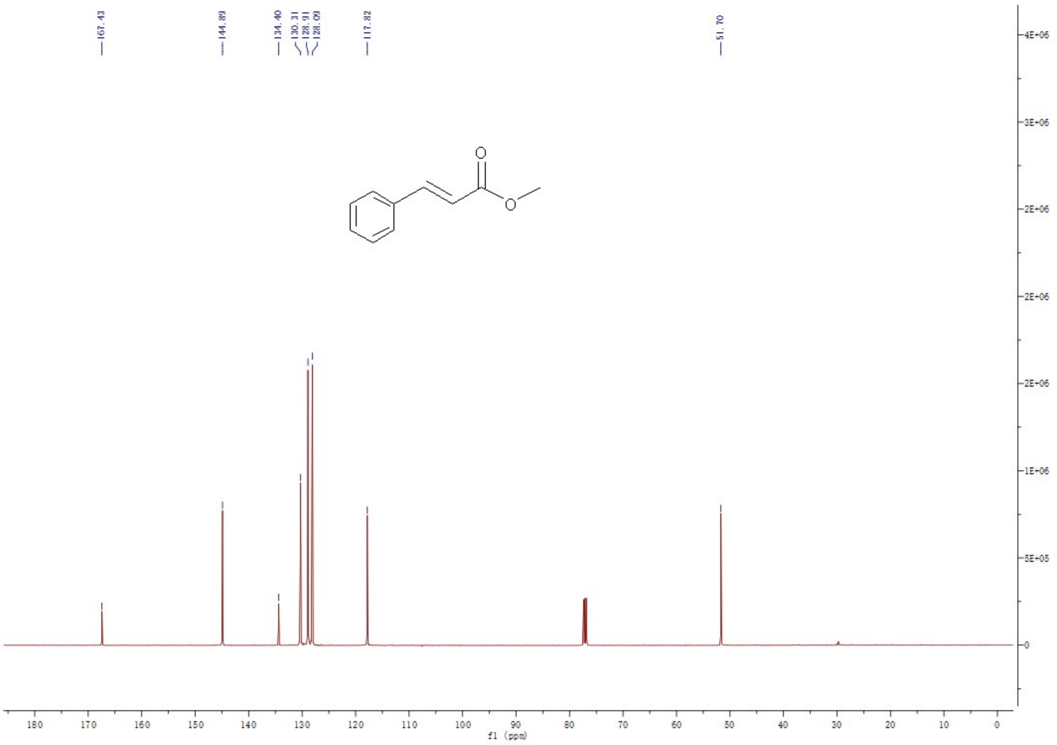

Compound **3j:** ^{1}H NMR (400 MHz, $CDCl_3$) δ 7.64 (d, *J* = 16.0 Hz, 1H), 7.27 (d, *J* = 6.2 Hz, 2H), 7.22 (t, *J* = 7.8 Hz, 1H), 7.14 (d, *J* = 7.4 Hz, 1H), 6.40 (d, *J* = 16.0 Hz, 1H), 3.76 (s, 3H), 2.31 (s, 3H); ^{13}C NMR (101 MHz, $CDCl_3$) δ 167.24, 144.90, 138.38, 134.29, 131.04, 128.67, 125.19, 117.49, 51.42, 21.13; IR (KBr) 2950, 1720, 1638, 1315, 1237, 1163, 1038, 982, 787, 681.

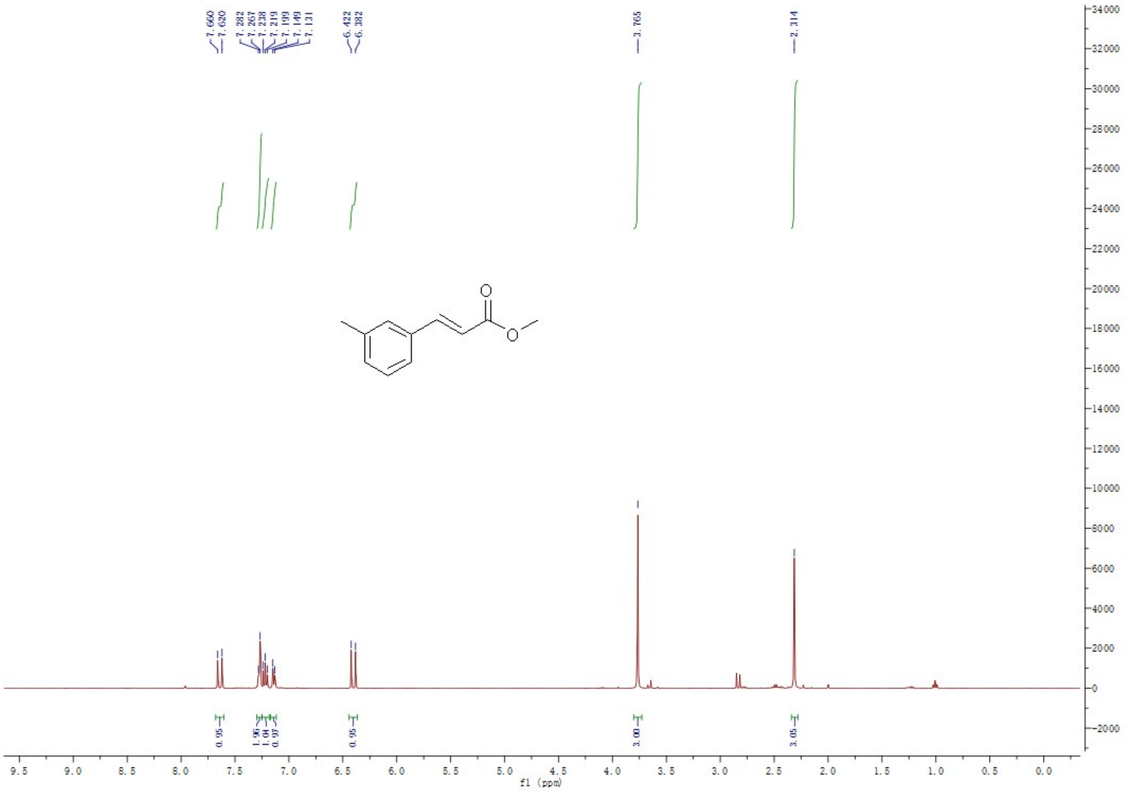

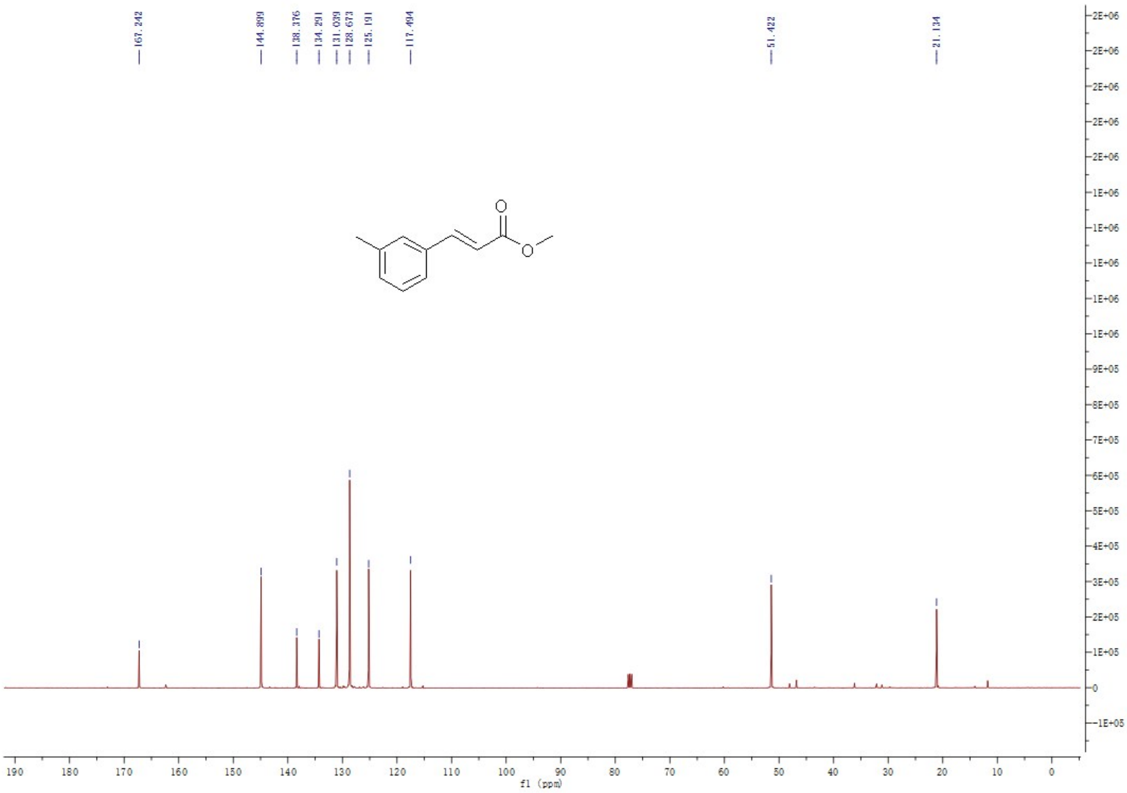

Compound **3k:** ^{1}H NMR (500 MHz, $CDCl_3$) δ 7.68 (d, J = 16.0 Hz, 1H), 7.52 (dd, J = 6.6, 2.8 Hz, 2H), 7.40 – 7.35 (m, 3H), 6.44 (d, J = 16.0 Hz, 1H), 4.21 (t, J = 6.7 Hz, 2H), 1.69 (dt, J = 14.6, 6.8 Hz, 2H), 1.49 – 1.39 (m, 2H), 0.97 (t, J = 7.4 Hz, 3H); ^{13}C NMR (126 MHz, $CDCl_3$) δ 167.11, 144.55, 134.47, 130.20, 128.86, 128.04, 118.29, 64.43, 30.77, 19.20, 13.74; IR (KBr) 2959, 1712, 1638, 1310, 1202, 1170, 979, 864, 767, 684.

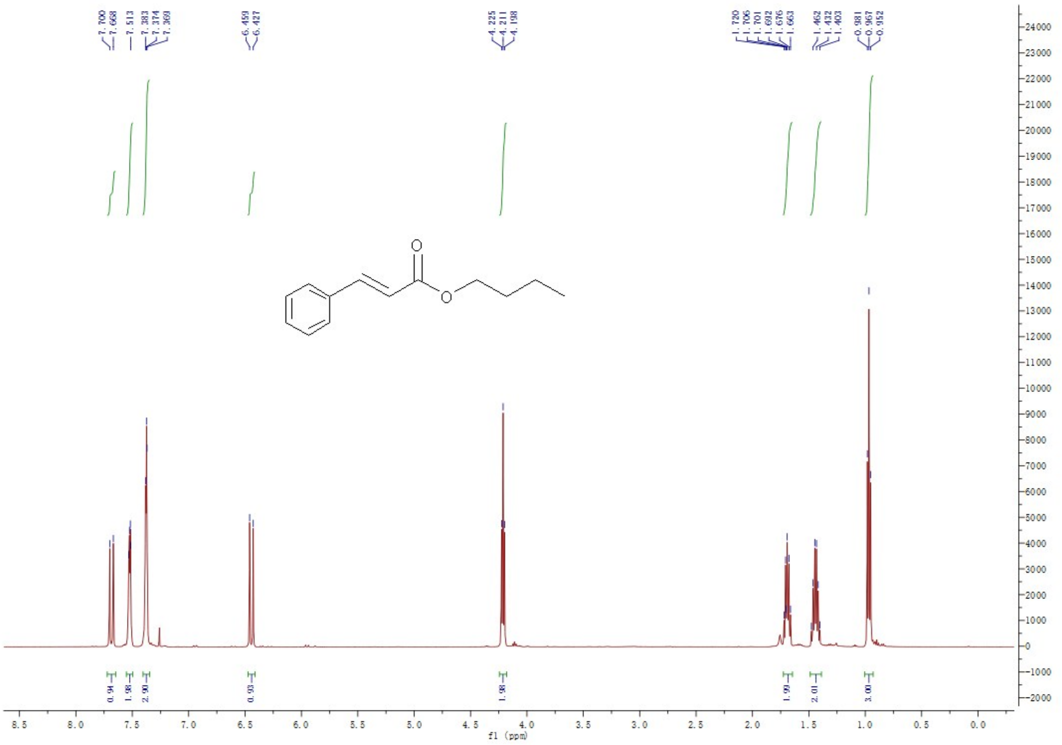

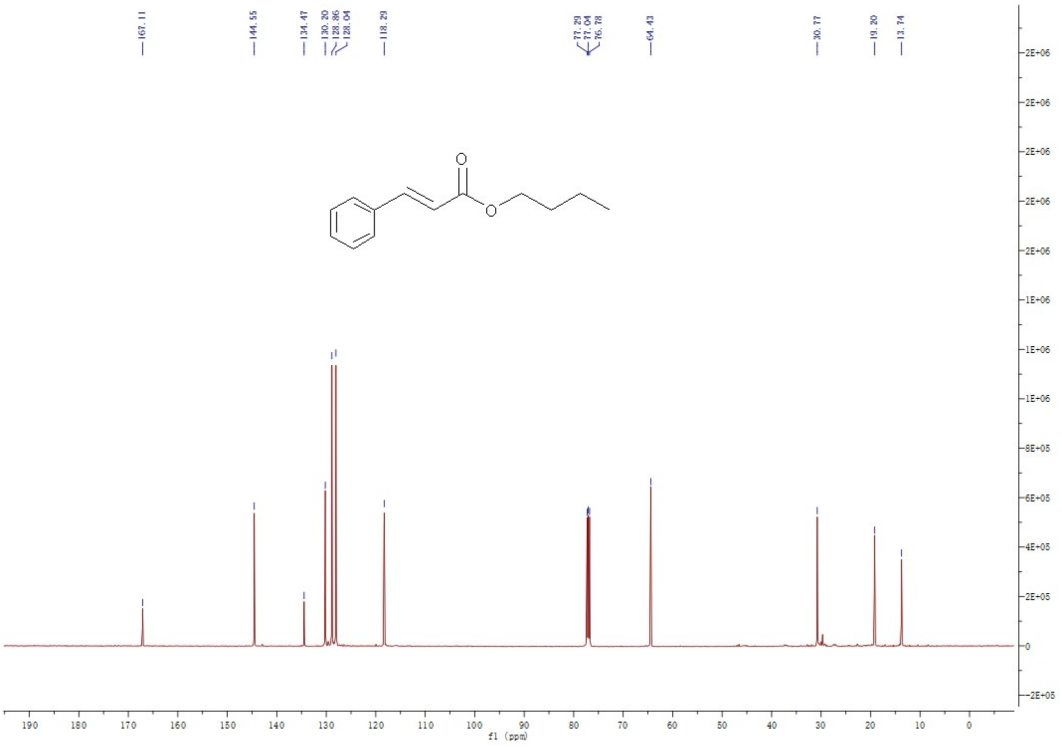

Compound **3l:** ^{1}H NMR (400 MHz, $CDCl_3$) δ 7.66 (d, *J* = 16.0 Hz, 1H), 7.33 (d, *J* = 7.2 Hz, 2H), 7.30 – 7.26 (m, 1H), 7.19 (d, *J* = 7.4 Hz, 1H), 6.43 (d, *J* = 16.0 Hz, 1H), 4.21 (t, *J* = 6.7 Hz, 2H), 2.37 (s, 3H), 1.75 – 1.64 (m, 2H), 1.50 – 1.37 (m, 2H), 0.97 (t, *J* = 7.4 Hz, 3H); ^{13}C NMR (101 MHz, $CDCl_3$) δ 167.15, 144.70, 138.50, 134.45, 131.00, 128.73, 128.69, 125.22, 118.09, 64.36, 30.78, 21.28, 19.19, 13.71; IR (KBr) 2959, 1713, 1638, 1456, 1311, 1236, 1175, 982, 861, 787, 681.

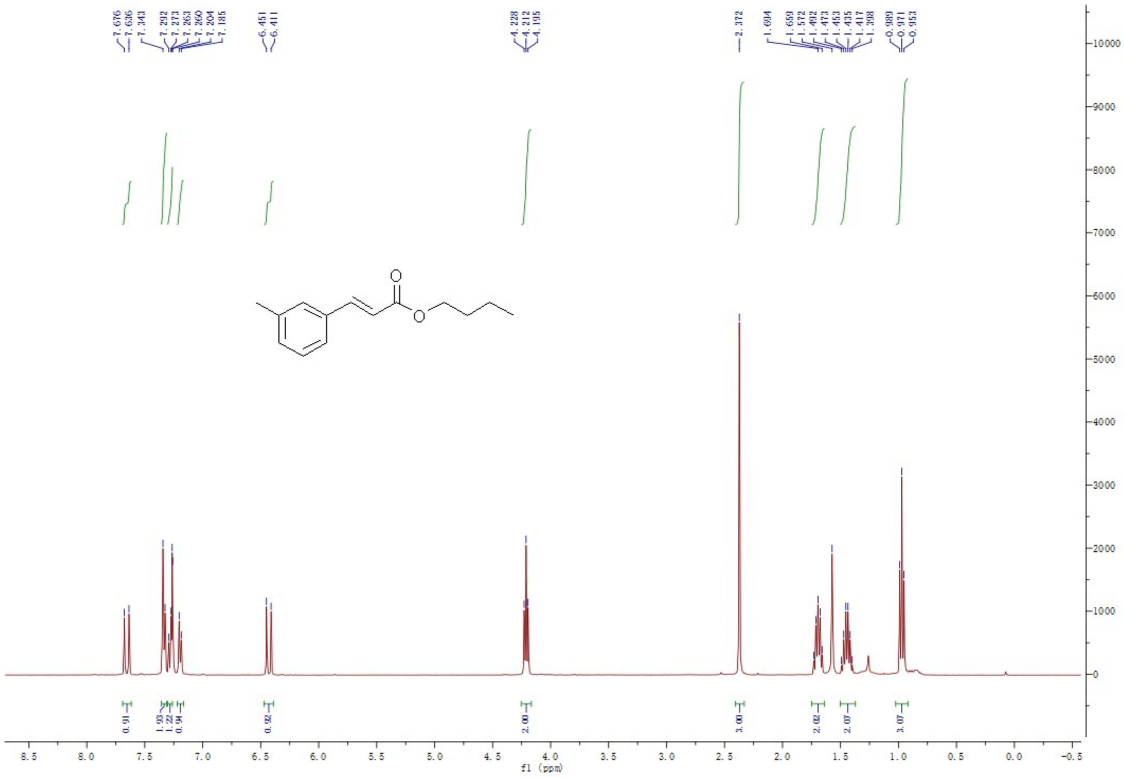

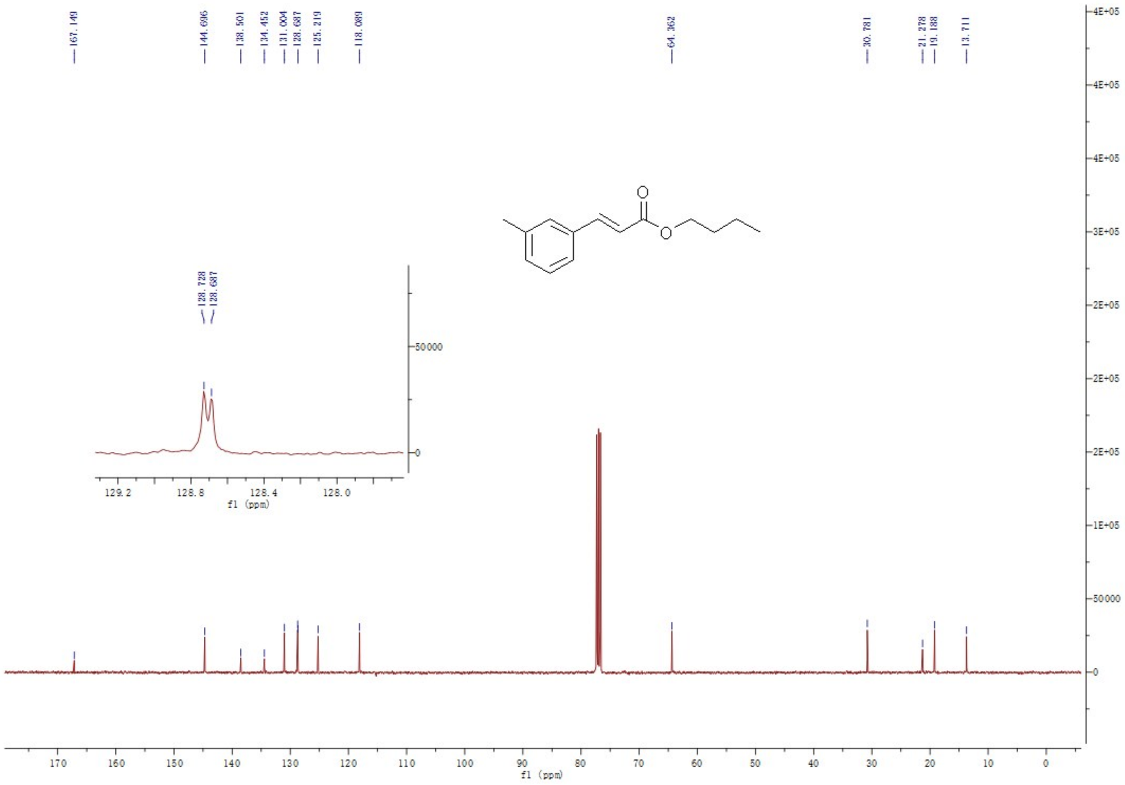

Compound **3m:** ^{1}H NMR (500 MHz, $CDCl_3$) δ 10.61 (s, 0H), 7.86 (d, *J* = 16.0 Hz, 1H), 7.61 (dd, *J* = 6.1, 2.5 Hz, 2H), 7.49 – 7.43 (m, 3H), 6.52 (d, *J* = 16.0 Hz, 1H); ^{13}C NMR (126 MHz, $CDCl_3$) δ 172.63, 147.12, 134.04, 130.77, 128.98, 128.40, 117.37; IR (KBr) 3026, 1676, 1629, 1421, 1312, 1285, 1222, 979, 768, 710, 684.

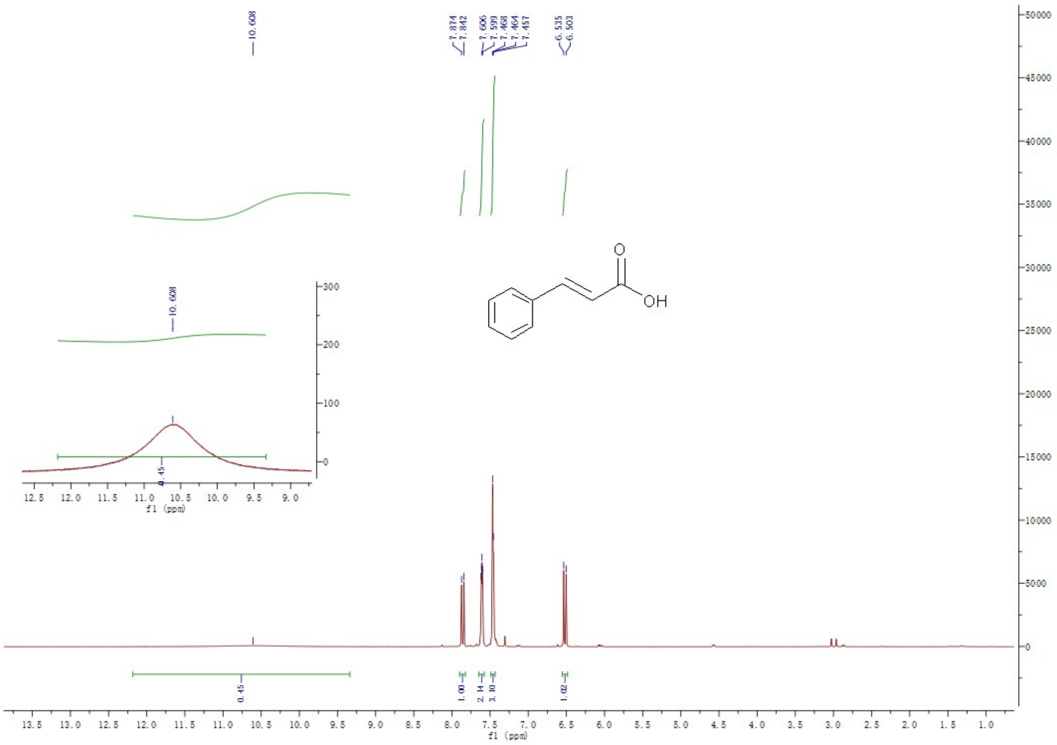

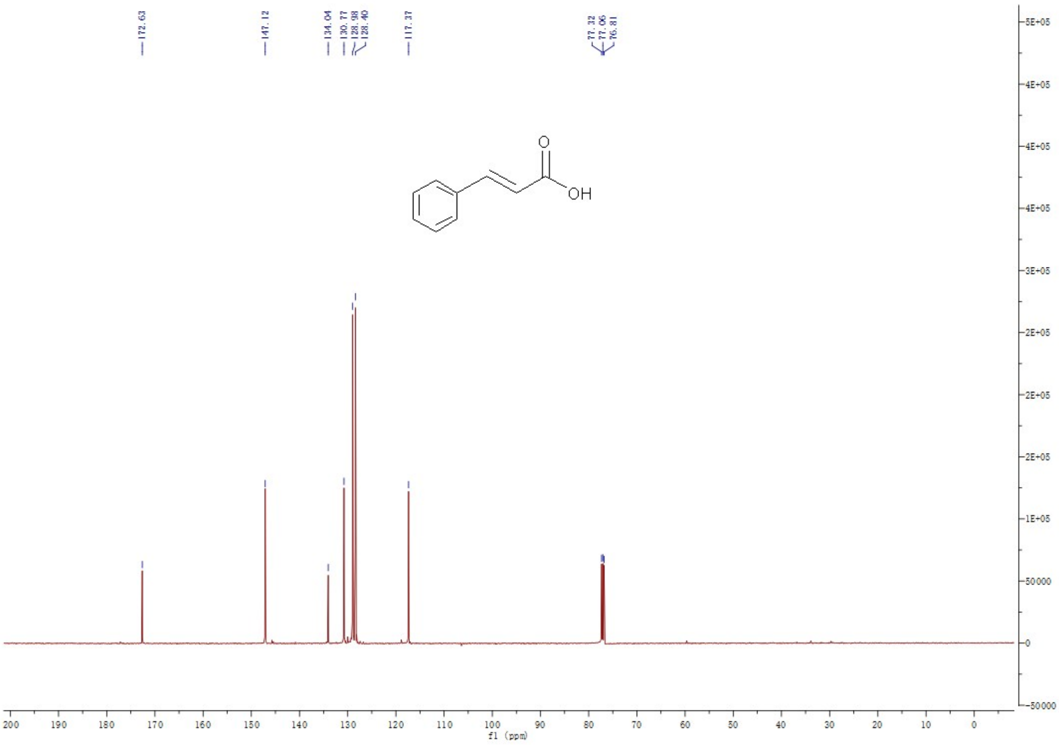

Compound **3n:** ^{1}H NMR (400 MHz, $CDCl_3$) δ 7.77 (d, J = 16.0 Hz, 1H), 7.45 (d, J = 8.0 Hz, 2H), 7.21 (d, J = 7.9 Hz, 2H), 6.41 (d, J = 15.9 Hz, 1H), 2.39 (s, 3H); ^{13}C NMR (101 MHz, $CDCl_3$) δ 172.03, 147.02, 141.23, 131.38, 129.68, 128.35, 116.12, 21.47; IR (KBr) 1688, 1577, 1422, 1273, 1160, 1010, 926, 849, 650.

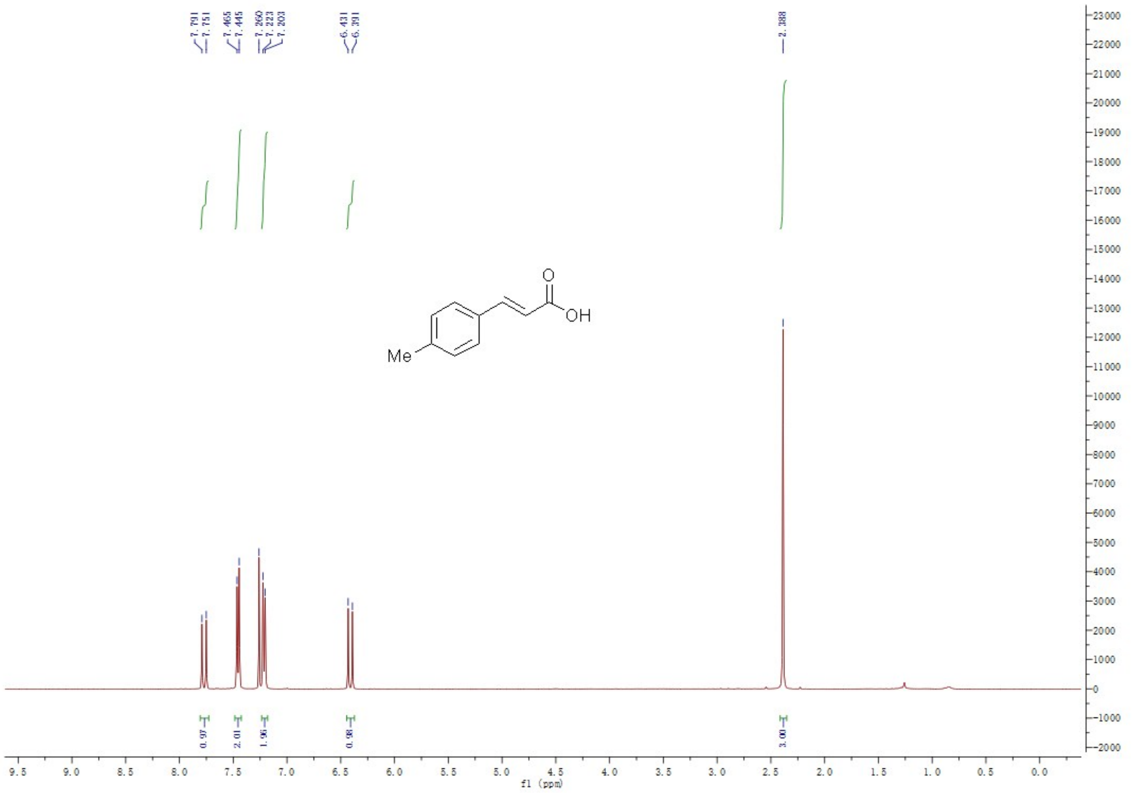

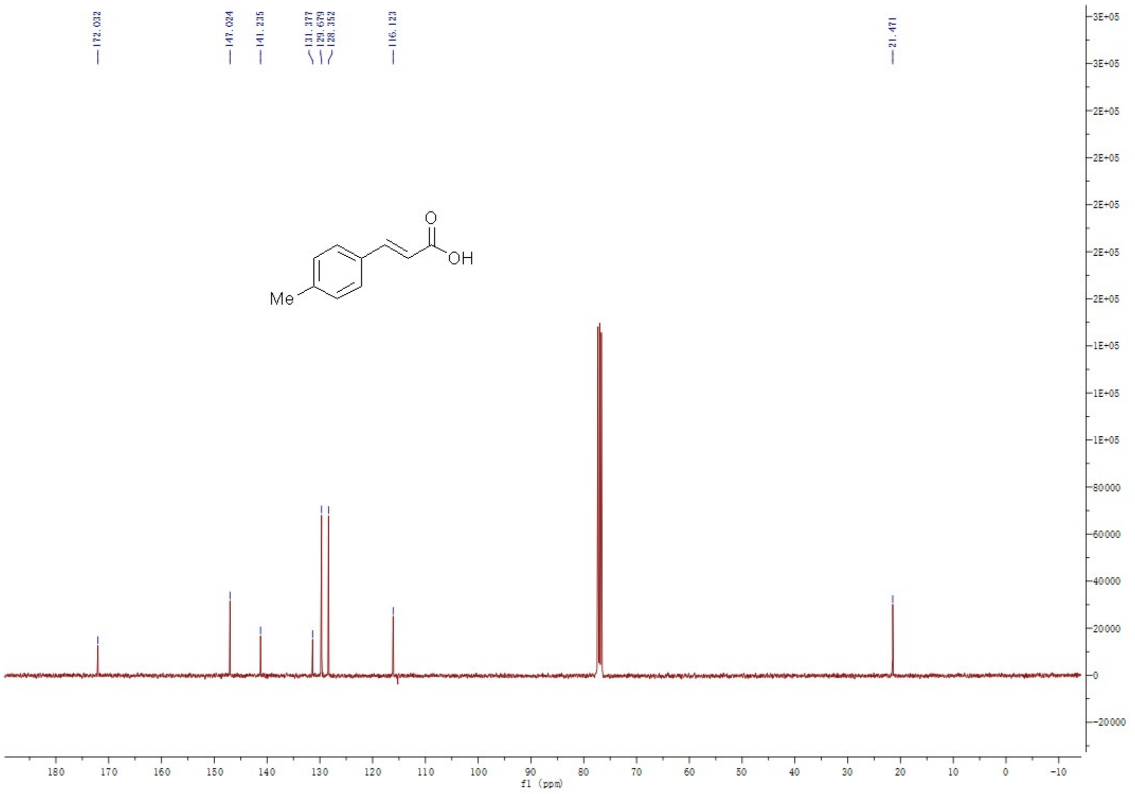

Compound **3o:** ^{1}H NMR (500 MHz, $CDCl_3$) δ 7.60 (s, 1H), 7.43 (d, J = 11.0 Hz, 2H), 6.98 (d, J = 11.0 Hz, 2H), 3.80 (s, 3H), 3.75 (s, 3H), 2.07 (s, 3H); ^{13}C NMR (126 MHz, $CDCl_3$) δ 168.74, 160.00, 138.48, 131.86, 128.12, 125.68, 114.33, 55.44, 55.06, 14.20; IR (KBr) 2951, 1709, 1605, 1511, 1303, 1253, 1177, 1119, 1033, 836, 750, 533.

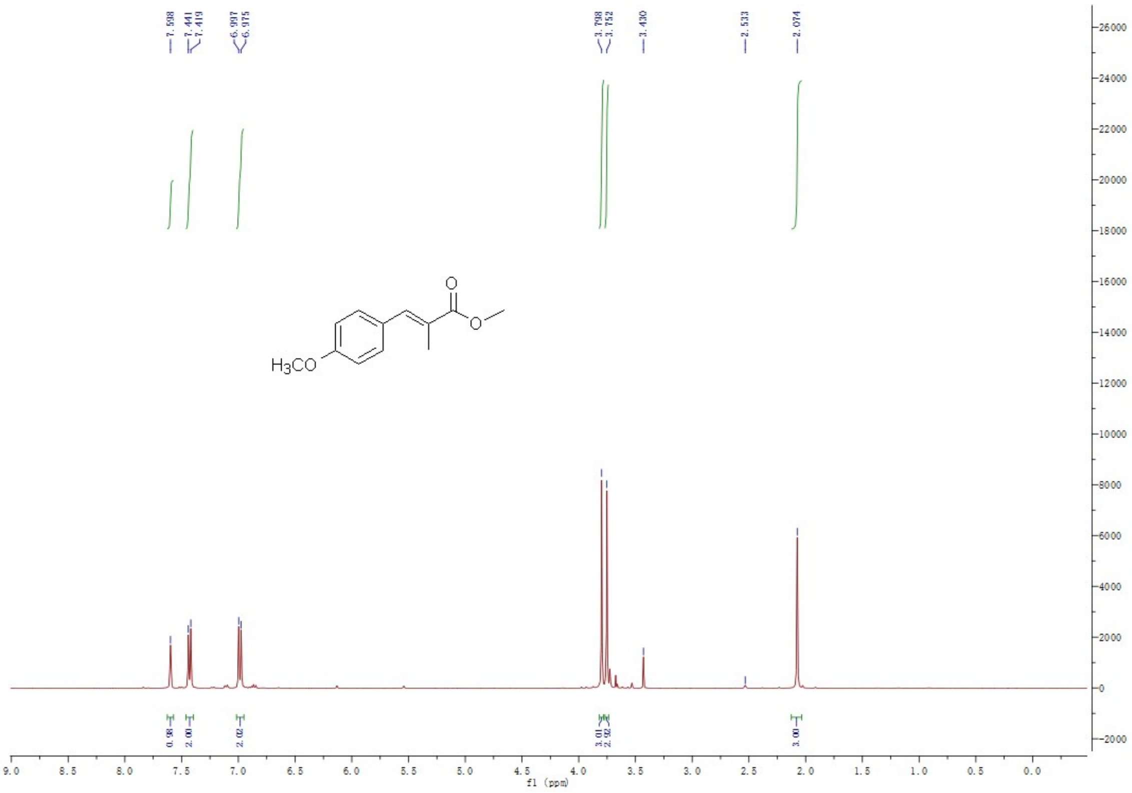

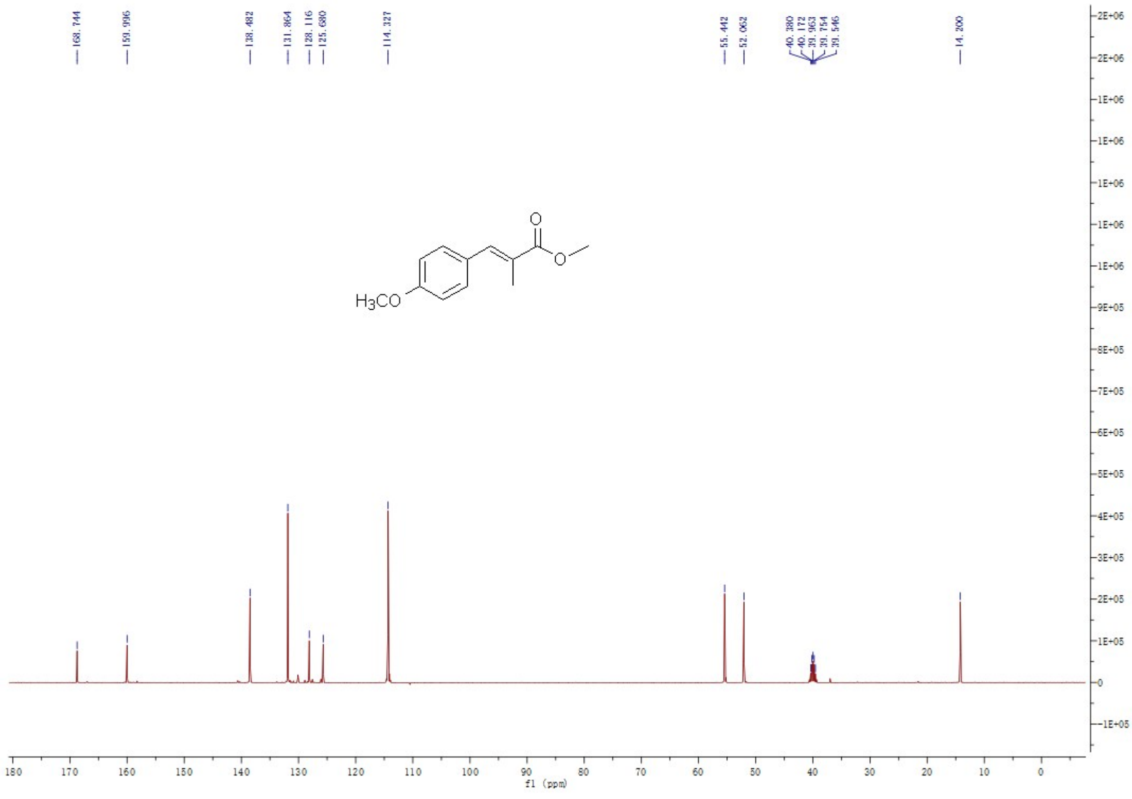

3) Typical procedure for the preparation of 5 (5a as example):

MOPs-Pd-I(50mg) was added to a solution of iodobenzene **1a** (2.5mmol), phenylboronic acid **4a** (3.0mmol), and K_3PO_4 (5.0mmol) in 10mL EtOH/H_2O (V_{EtOH}/V_{H2O} = 2:1). The mixture was stirred under a N_2 atmosphere at 80°C for 1.0 hour. After the substrate iodobenzene **1a** was consumed as indicated by TLC, the mixture was poured into ice-water (100mL) under stirring. The mixture was extracted with dichloromethane (3 × 20mL), and the combined organic phase was washed with water (3× 20mL), dried over $MgSO_4$, filtered and concentrated in vacuo. The crude product was purified by flash chromatography (silica gel, petroleum ether: diethyl ether = 20: 1) to give **5a** as a white solid (373 mg, 97%).

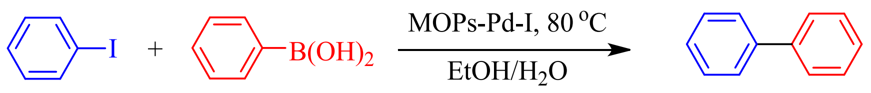

1a **4a** **5a**

The analytical data of **5** was reported by reference (X. Liu, W. Xu, D. Xiang, Z. Zhang, D. Chen, Y. Hu, Y. Li, Y. Ouyang and H. Lin, *New J. Chem.*, 2019, DOI: 10.1039/C9NJ02444A).

4) Analytical data and Copies of NMR spectra for 5

5a: 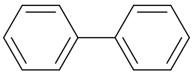

White solid: ^{1}H NMR (500 MHz, $CDCl_3$) δ 7.47 (d, J = 7.4 Hz, 2H), 7.31 (t, J = 7.6 Hz, 2H), 7.22 (t, J = 7.3 Hz, 1H); ^{13}C NMR (125 MHz, $CDCl_3$) δ = 127.3, 127.4, 128.9, 141.3; IR (KBr) 3034, 1479, 1429, 1170, 1007, 727, 696, 611.

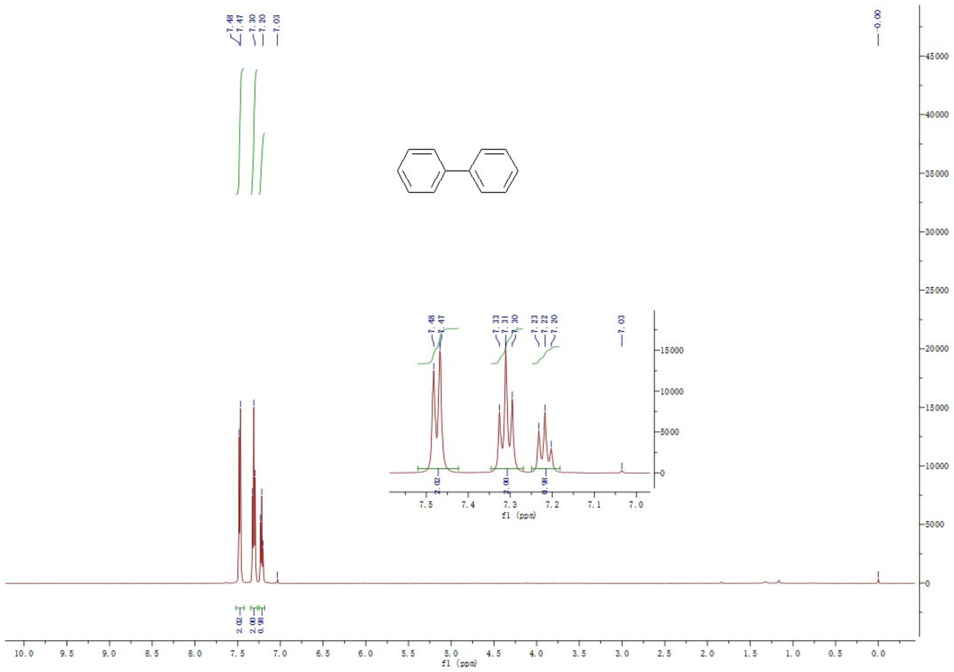

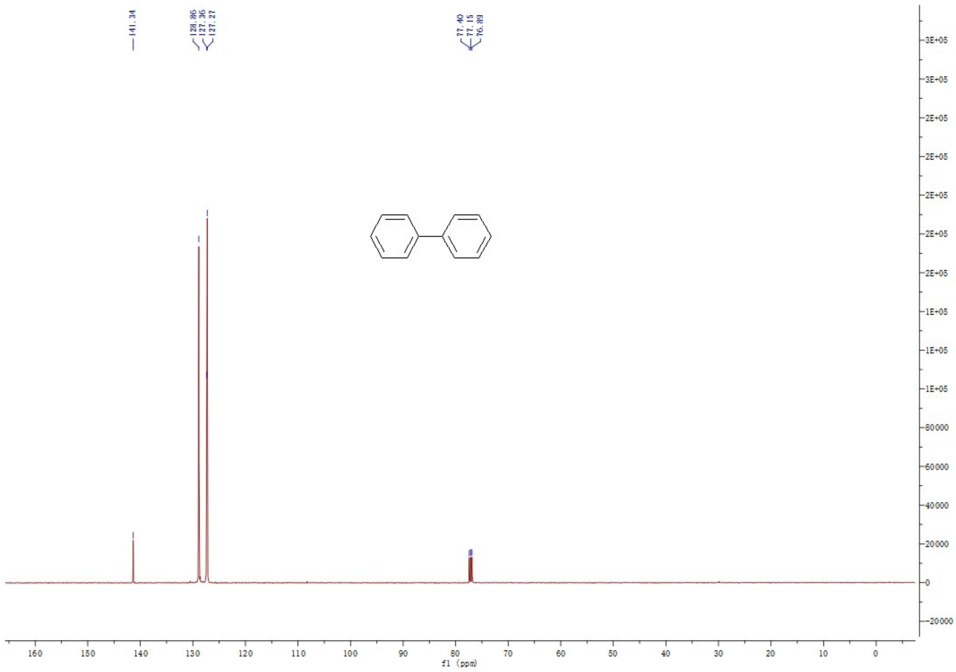

5b: 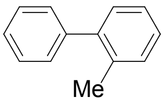

White solid: ^{1}H NMR (500 MHz, $CDCl_3$) δ = 7.57-7.52 (m, 2H), 7.48 (dd, J = 7.1, 5.4 Hz, 3H), 7.43-7.36 (m, 4H), 2.42 (s, 3H); ^{13}C NMR (125 MHz, $CDCl_3$) δ = 20.6, 125.9, 126.9, 128.2, 129.3, 129.9, 130.4, 135.4, 142.0, 142.1; IR (KBr) 3020, 1599, 1479, 1341, 1009, 726, 701, 617.

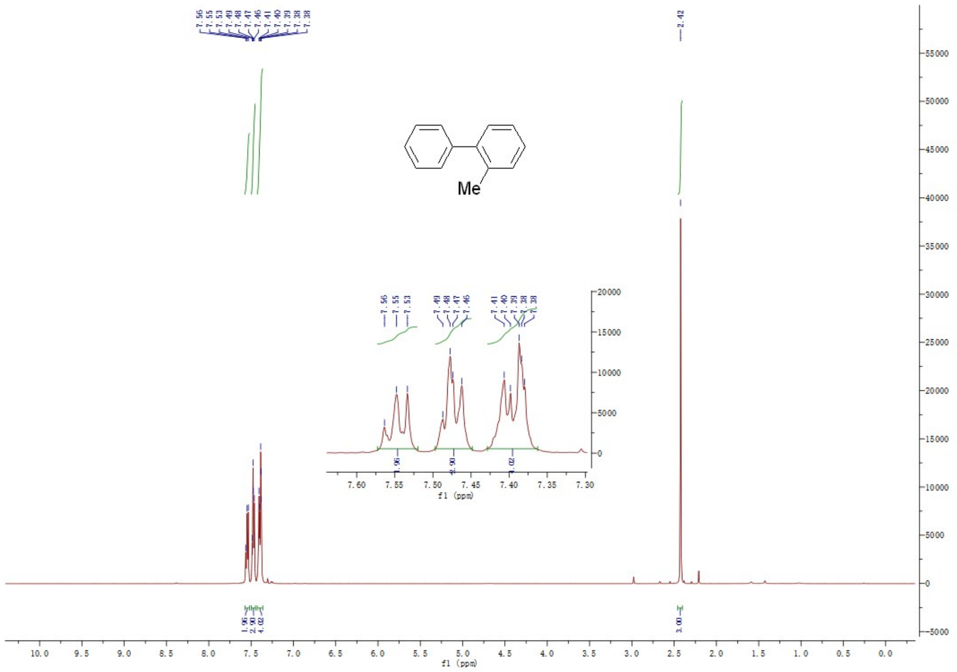

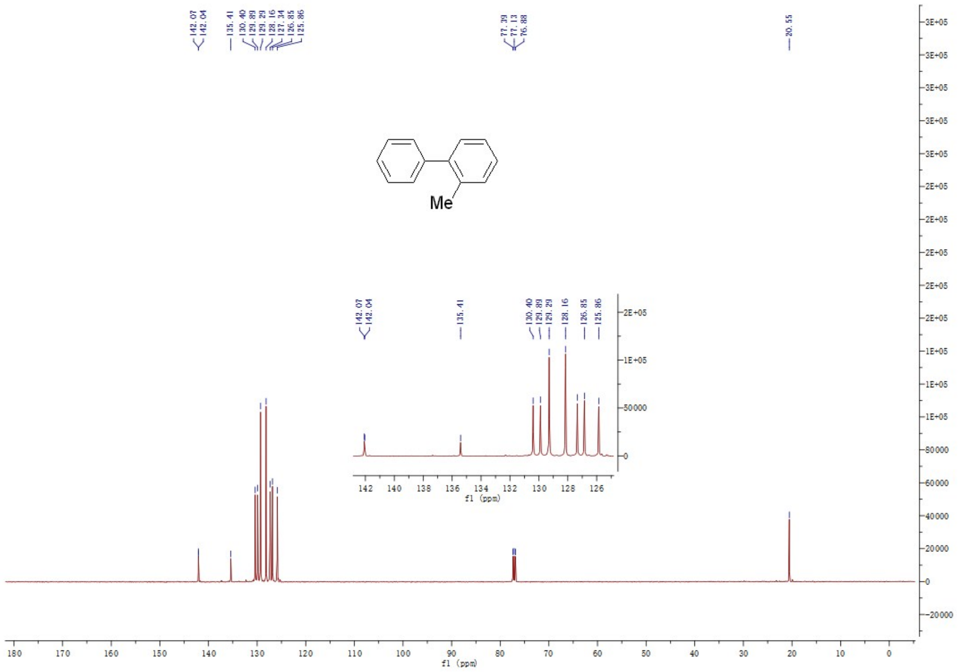

5c: 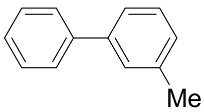

White solid: ^{1}H NMR (500 MHz, $CDCl_3$) δ = 7.70 (d, *J* = 7.5 Hz, 2H), 7.53 (dd, *J* = 16.4, 8.7 Hz, 4H), 7.44 (t, *J* = 7.5 Hz, 2H), 7.27 (d, *J* = 7.3 Hz, 1H), 2.53 (s, 3H); ^{13}C NMR (125 MHz, $CDCl_3$) δ = 21.6, 124.3, 127.2, 128.0, 128.1, 128.8, 138.4, 141.3, 141.4; IR (KBr) 3029, 1600, 1481, 791, 752, 697, 616.

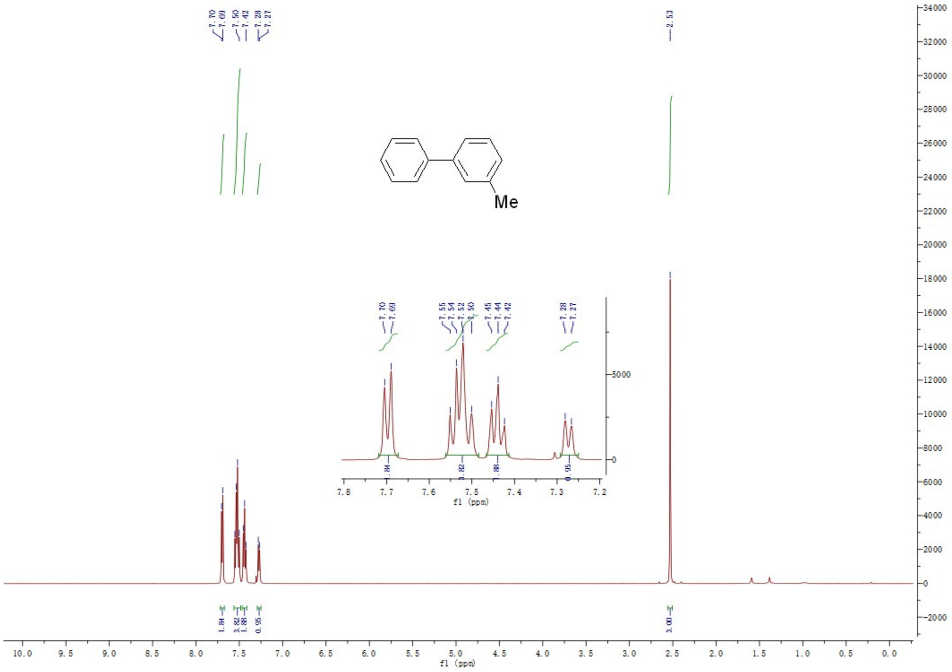

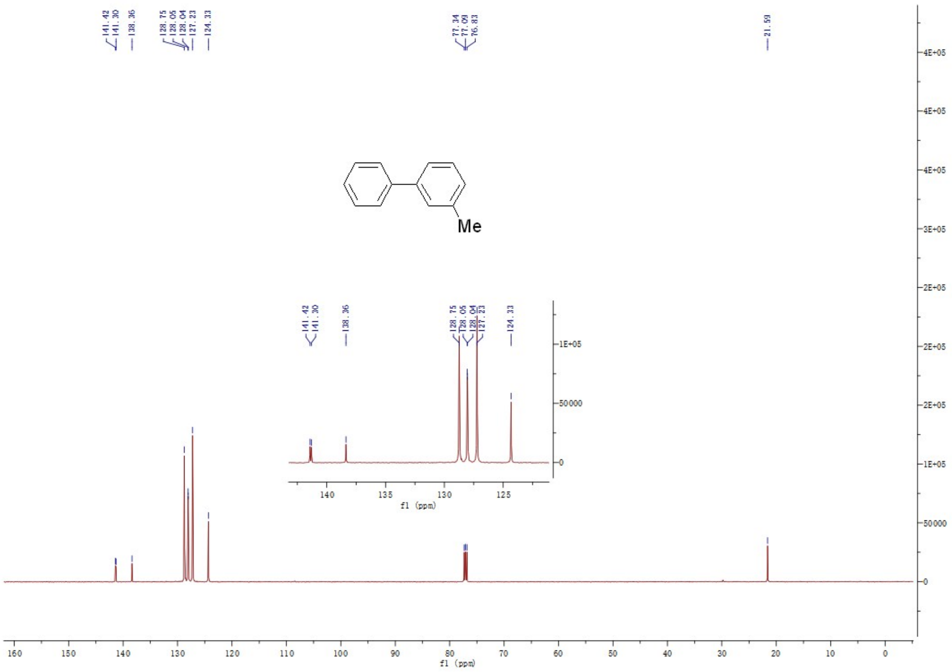

5d: 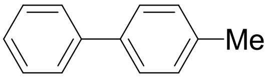

White solid: ^{1}H NMR (500 MHz, $CDCl_3$) δ = 7.71 (d, *J* = 7.3 Hz, 2H), 7.63 (d, *J* = 8.0 Hz, 2H), 7.55 (t, *J* = 7.7 Hz, 2H), 7.45 (t, *J* = 7.4 Hz, 1H), 7.38 (d, *J* = 7.9 Hz, 2H), 2.53 (s, 3H); ^{13}C NMR (125 MHz, $CDCl_3$) δ = 21.6, 124.3, 127.2, 128.0, 128.1, 128.8, 138.4, 141.3, 141.4; IR (KBr) 2917, 1487, 1377, 1006, 822, 754, 689.

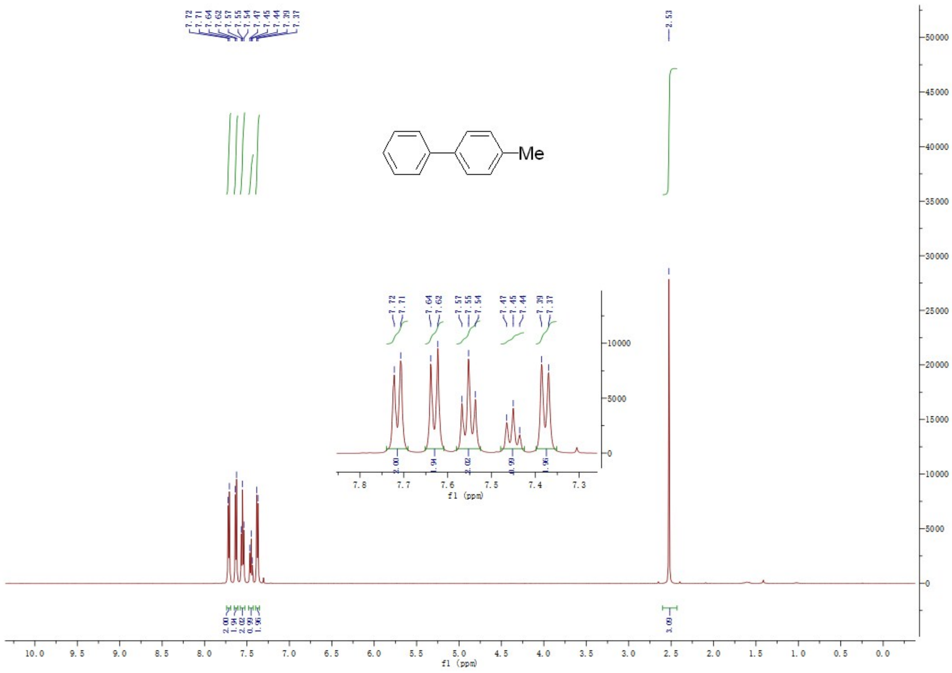

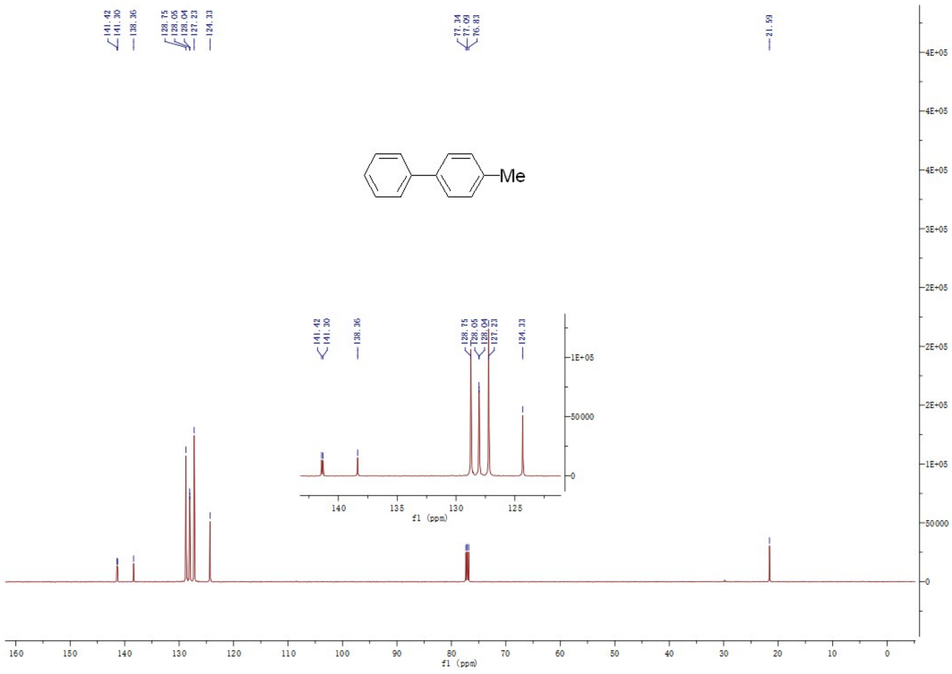

5e: 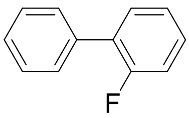

White solid: ^{1}H NMR (500 MHz, $CDCl_3$) δ = 7.67 (d, *J* = 7.8 Hz, 2H), 7.55 (dd, *J* = 10.4, 4.7 Hz, 3H), 7.47 (t, *J* = 7.3 Hz, 1H), 7.43-7.38 (m, 1H), 7.30 (t, *J* = 7.4 Hz, 1H), 7.28-7.23 (m, 1H); ^{13}C NMR (125 MHz, $CDCl_3$) δ = 124.4, 127.7, 128.5, 128.9, 129.0, 129.1, 130.8, 135.9, 158.6, 160.8; IR (KBr) 2924, 1476, 1435, 1202, 1106, 727, 698, 611.

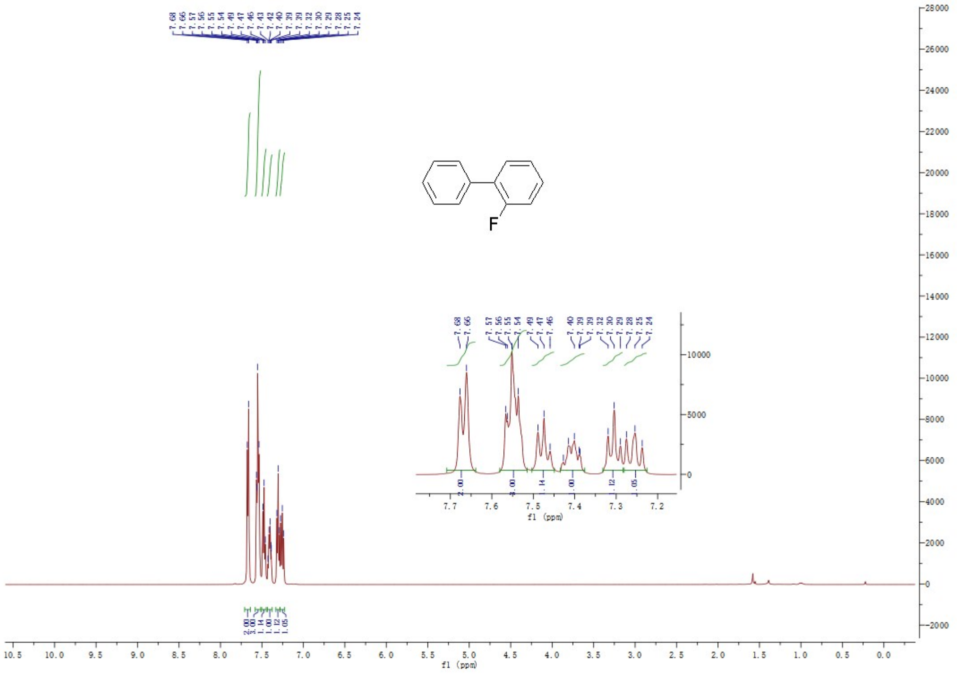

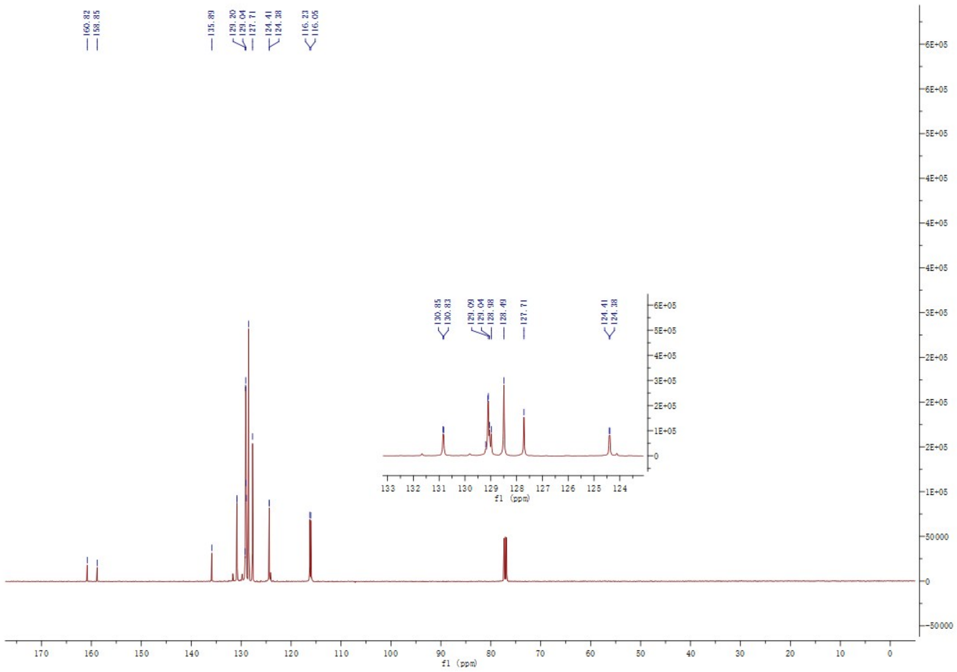

5f: 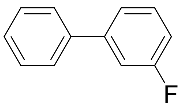

White solid: ^{1}H NMR (500 MHz, $CDCl_3$) δ = 7.67 (d, *J* = 7.3 Hz, 2H), 7.54 (t, *J* = 7.6 Hz, 2H), 7.50-7.44 (m, 3H), 7.39 (dd, *J* = 11.6, 1.4 Hz, 1H), 7.14 (ddd, *J* = 8.8, 5.1, 2.3 Hz, 1H); ^{13}C NMR (125 MHz, $CDCl_3$) δ = 140.1, 122.8, 127.2, 127.9, 128.9, 130.2, 140.0, 143.5, 162.3, 164.2; IR (KBr) 1589, 1479, 1422, 1290, 1185, 1077, 877, 756, 695, 613.

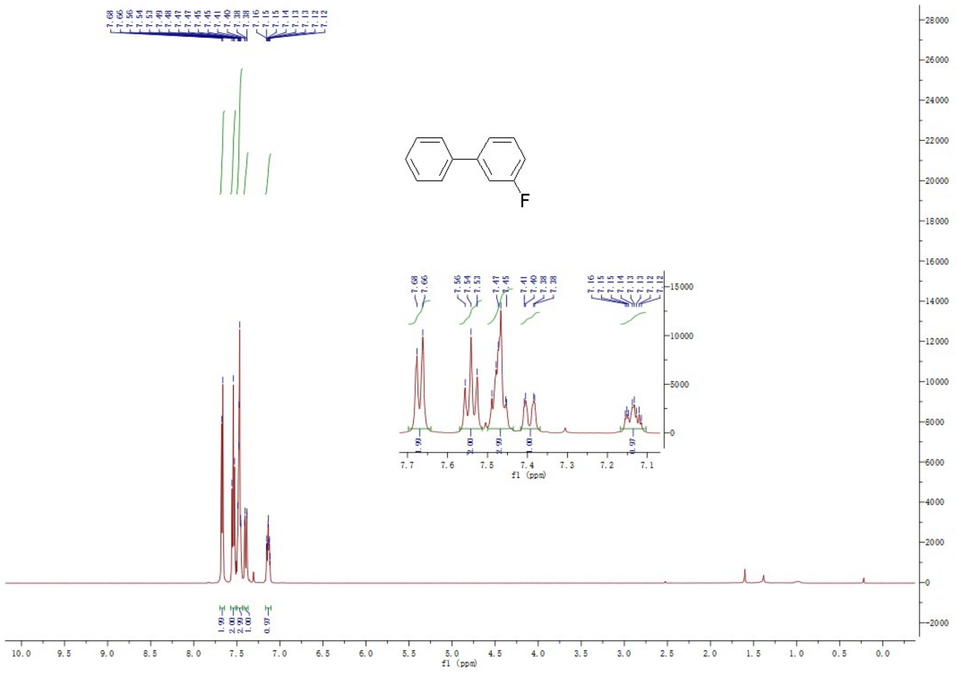

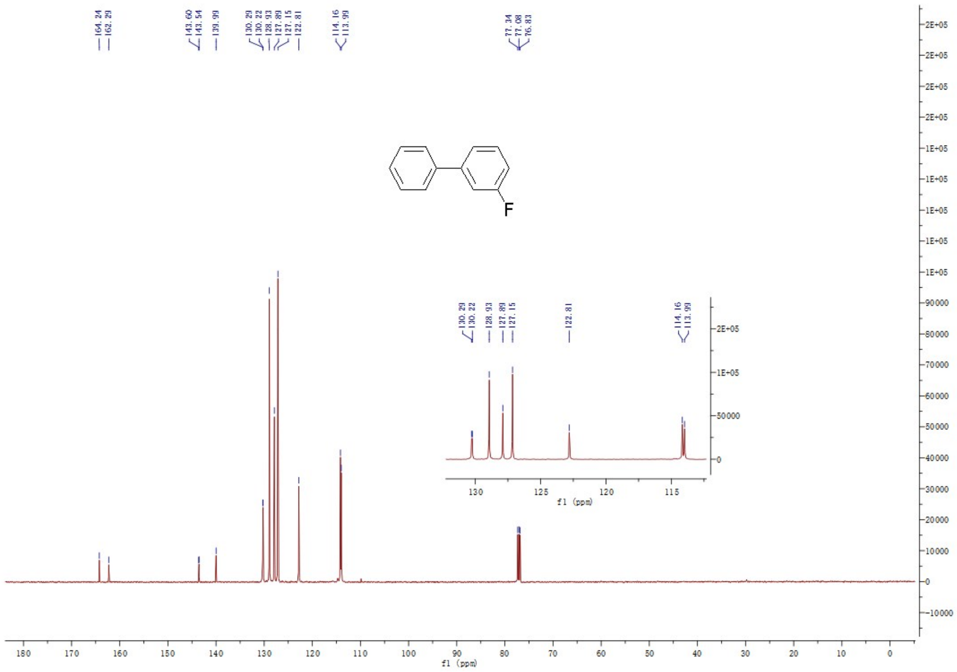

5g: 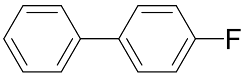

White solid: ^{1}H NMR (500 MHz, $CDCl_3$) δ = 7.65 (t, *J* = 6.5 Hz, 4H), 7.55 (t, *J* = 7.5 Hz, 2H), 7.49-7.44 (m, 1H), 7.24 (t, *J* = 8.5 Hz, 2H); ^{13}C NMR (125 MHz, $CDCl_3$) δ = 115.7, 127.1, 127.3, 128.8, 137.4, 140.3, 161.6, 163.5; IR (KBr) 1598, 1519, 1484, 1236, 1195, 1006, 837, 758, 687.

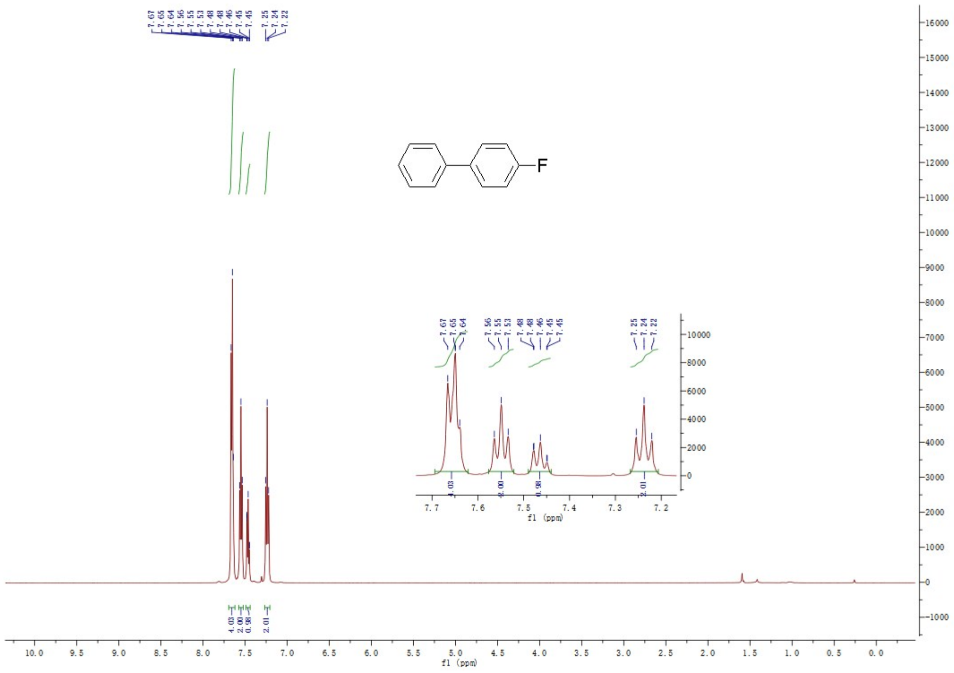

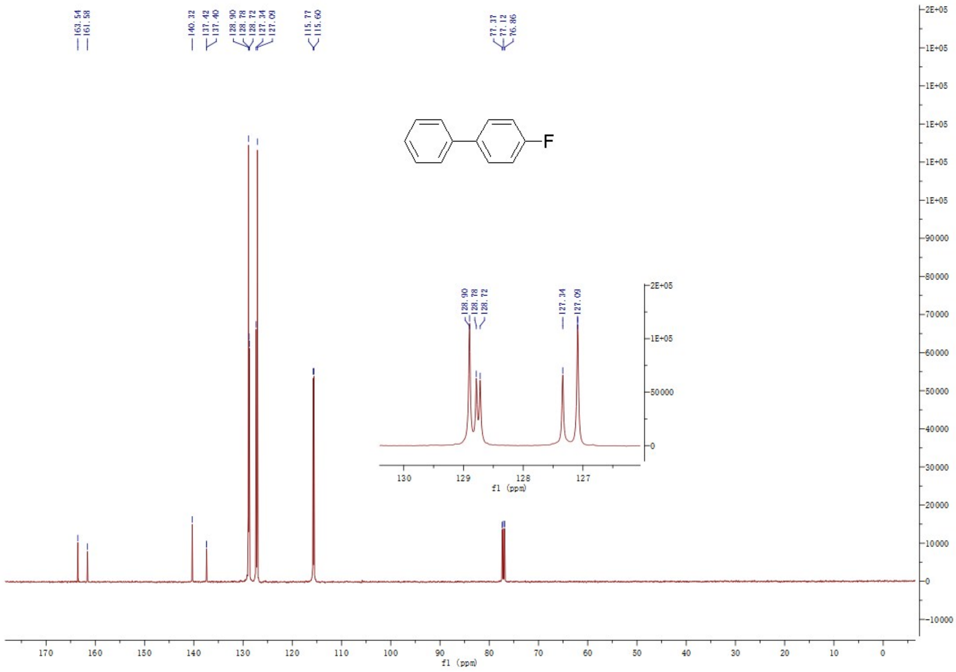

5h: 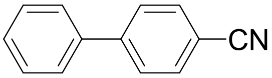

White solid: ^{1}H NMR (500 MHz, $CDCl_3$) δ = 7.57-7.52 (m, 2H), 7.48 (dd, *J* = 7.1, 5.4 Hz, 3H), 7.43-7.36 (m, 4H), 2.42 (s, 3H); ^{13}C NMR (125 MHz, $CDCl_3$) δ = 110.9, 118.9, 127.2, 127.7, 128.7, 129.1, 132.6, 139.2, 145.7; IR (KBr) 2965, 2226, 1605, 1484, 1261, 1077, 848, 769, 697.

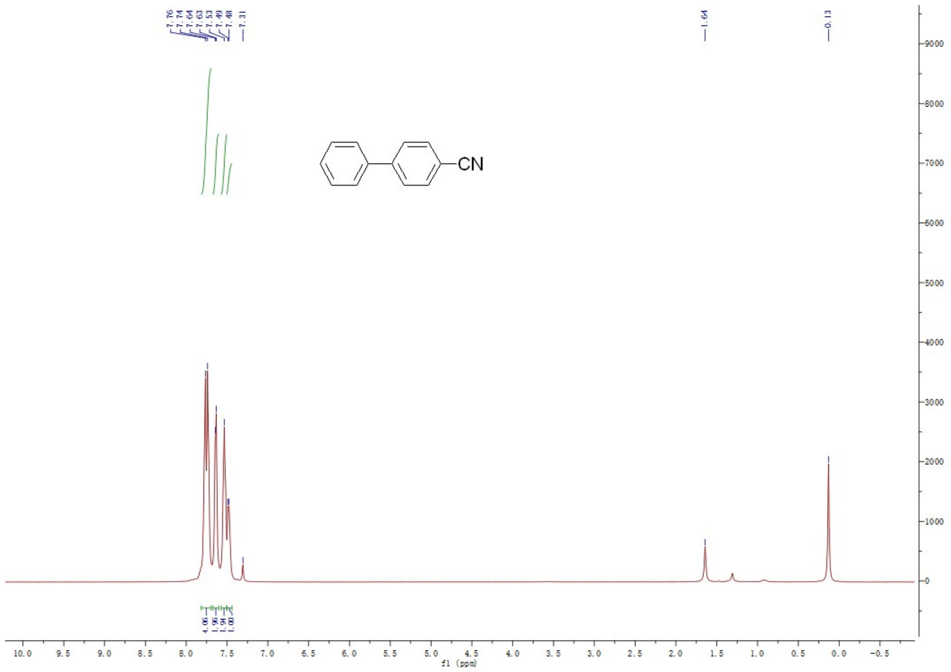

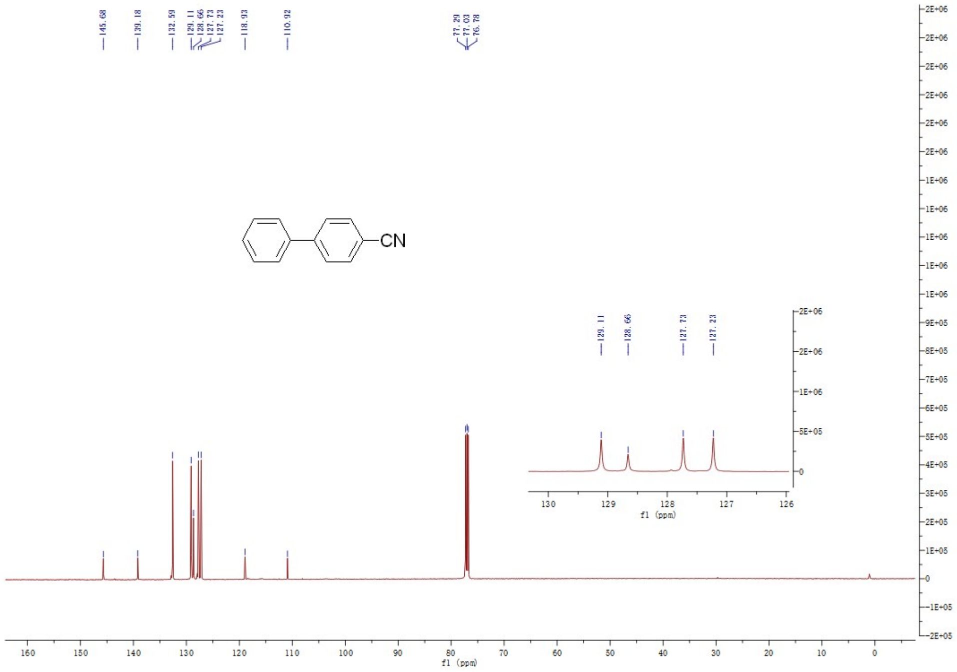

5i: 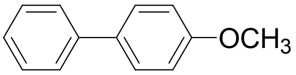

White solid: ^{1}H NMR (500 MHz, $CDCl_3$) δ = 7.63 (dd, *J* = 11.5, 8.2 Hz, 4H), 7.50 (t, *J* = 7.6 Hz, 2H), 7.39 (t, *J* = 7.3 Hz, 1H), 7.06 (d, *J* = 8.7 Hz, 2H), 3.92 (s, 3H); ^{13}C NMR (125 MHz, $CDCl_3$) δ = 55.4, 114.3, 126.7, 126.8, 128.2, 128.7, 133.8, 140.9, 159.2; IR (KBr) 1605, 1485, 1248, 1199, 1036, 834, 760, 688.

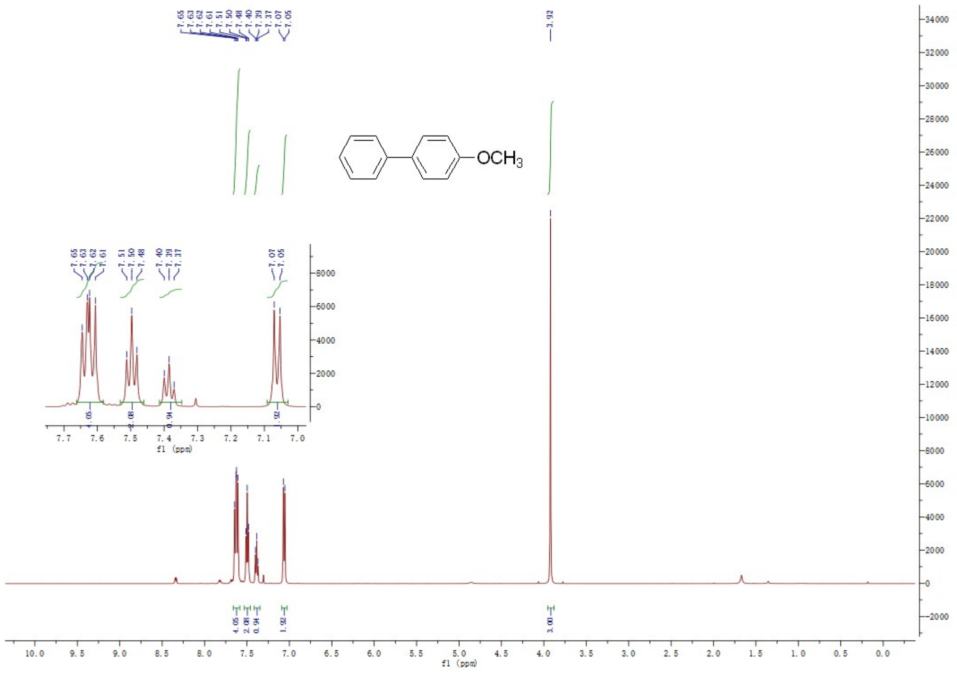

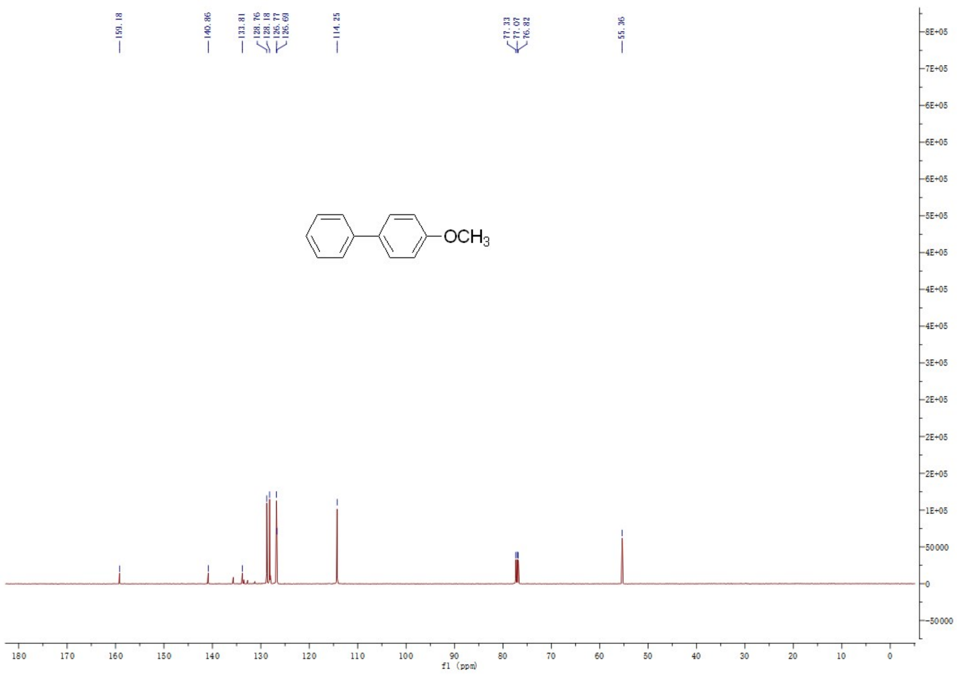

Supplement: RA-009-C9RA07303E-s001 [file RA-009-C9RA07303E-s001.pdf]
